# Fluorescently Labeled PLGA Nanoparticles for Visualization In Vitro and In Vivo: The Importance of Dye Properties

**DOI:** 10.3390/pharmaceutics13081145

**Published:** 2021-07-27

**Authors:** Vasilisa Zhukova, Nadezhda Osipova, Aleksey Semyonkin, Julia Malinovskaya, Pavel Melnikov, Marat Valikhov, Yuri Porozov, Yaroslav Solovev, Pavel Kuliaev, Enqi Zhang, Bernhard A. Sabel, Vladimir Chekhonin, Maxim Abakumov, Alexander Majouga, Jörg Kreuter, Petra Henrich-Noack, Svetlana Gelperina, Olga Maksimenko

**Affiliations:** 1 Drug Delivery Systems Laboratory, D. Mendeleev University of Chemical Technology of Russia, Miusskaya pl. 9, 125047 Moscow, Russia; vassaz@list.ru (V.Z.); kompacc@yandex.ru (N.O.); semyonkin.aleksey@gmail.com (A.S.); j.malinowskaya@gmail.com (J.M.); abakumov1988@gmail.com (M.A.); rector@muctr.ru (A.M.); svetlana.gelperina@gmail.com (S.G.); 2 Department of Neurobiology, V. Serbsky Federal Medical Research Centre of Psychiatry and Narcology of the Ministry of Health of the Russian Federation, Kropotkinskiy per. 23, 119034 Moscow, Russia; proximopm@gmail.com (P.M.); marat.valikhov@gmail.com (M.V.); chekhoninnew@yandex.ru (V.C.); 3 World-Class Research Center “Digital Biodesign and Personalized Healthcare”, I.M. Sechenov First Moscow State Medical University, Trubetskaya ul. 8, 119048 Moscow, Russia; yuri.porozov@gmail.com; 4 Department of Computational Biology, Sirius University of Science and Technology, Olympic Ave 1, 354340 Sochi, Russia; 5 Laboratory of Bioinformatics Approaches in Combinatorial Chemistry and Biology, Department of Functioning of Living Systems, Institute of Bioorganic Chemistry, Russian Academy of Sciences, Miklukho-Maklaya ul. 16/10, 119991 Moscow, Russia; solovev@ibch.ru; 6 TheoMAT Group, ITMO University, Kronverkskiy pr. 49, 197101 Saint Petersburg, Russia; kulyaevp@gmail.com; 7 Institute of Medical Psychology, Otto-von-Guericke University, Leipziger Str. 44, 39120 Magdeburg, Germany; enqi.zhang@med.ovgu.de (E.Z.); bernhard.sabel@med.ovgu.de (B.A.S.); phnoack@gmail.com (P.H.-N.); 8 Department of Medical Nanobiotecnology, Pirogov Russian National Research Medical University, Ostrovityanova ul 1, 117997 Moscow, Russia; 9 Institute of Pharmaceutical Technology, Biocenter, Goethe University, Max-von-Laue-Str. 9, 60438 Frankfurt am Main, Germany; Kreuter@em.uni-frankfurt.de; 10 Clinic of Neurology with Institute of Translational Neurology, University Clinic Muenster, Mendel Str. 7, 48149 Muenster, Germany

**Keywords:** PLGA nanoparticles, fluorescent labeling, DiI, coumarin 6, rhodamine 123, Cy5.5, quantum yield, brightness, stability of fluorescent label, confocal microscopy, intracellular internalization, in vivo neuroimaging

## Abstract

Fluorescently labeled nanoparticles are widely used for evaluating their distribution in the biological environment. However, dye leakage can lead to misinterpretations of the nanoparticles’ biodistribution. To better understand the interactions of dyes and nanoparticles and their biological environment, we explored PLGA nanoparticles labeled with four widely used dyes encapsulated (coumarin 6, rhodamine 123, DiI) or bound covalently to the polymer (Cy5.5.). The DiI label was stable in both aqueous and lipophilic environments, whereas the quick release of coumarin 6 was observed in model media containing albumin (42%) or liposomes (62%), which could be explained by the different affinity of these dyes to the polymer and lipophilic structures and which we also confirmed by computational modeling (log PDPPC/PLGA: DiI—2.3, Cou6—0.7). The importance of these factors was demonstrated by in vivo neuroimaging (ICON) of the rat retina using double-labeled Cy5.5/Cou6-nanoparticles: encapsulated Cou6 quickly leaked into the tissue, whereas the stably bound Cy.5.5 label remained associated with the vessels. This observation is a good example of the possible misinterpretation of imaging results because the coumarin 6 distribution creates the impression that nanoparticles effectively crossed the blood–retina barrier, whereas in fact no signal from the core material was found beyond the blood vessels.

## 1. Introduction

Polymeric nanoparticles hold promise as carriers for the targeted delivery of diagnostics or drugs. By altering the biodistribution of drugs, nanoparticles can improve their therapeutic efficacy and reduce adverse side effects. Therefore, comprehensive knowledge of a nanocarrier biodistribution profile is of paramount importance for the successful development of delivery systems [1]. Radioactive labeling is probably an unsurpassable technique for the quantitative analysis of bulk nanoparticle uptake in the organs, using, for example, single-photon emission computed tomography (SPECT) or positron emission tomography (PET). However, besides the extra efforts necessary for synthesizing and handling the radioactive marker substances, this approach offers little information regarding the precise localization of nanocarriers in target tissues and cells due to the intrinsically limited spatial resolution [2,3]. This information can, however, be obtained by fluorescence imaging. Because of the availability of highly sensitive imaging techniques, such as laser confocal fluorescent microscopy, flow cytometry and systems for the in vivo imaging of fluorescence, fluorescently labeled nanoparticles have become increasingly important in biomedical research (reviewed in several papers [4,5,6,7,8,9]).

Polymeric nanocarriers used for drug delivery are usually not fluorescent per se, and therefore their visualization in a biological environment requires labeling with fluorescent dyes. According to Fili and Toseland, an ideal “fluorescent label should be small, bright and stable, without causing any perturbation to the biological system [10].” The original phrase refers to the labeling of biological molecules; however, the same principle can be applied to the labeling of nanoparticles. These requirements can be fulfilled by certain small-molecule organic dyes. Obviously, fluorescently labeled nanoparticles serving as a prototype for preclinical evaluation of the nanoparticulate formulation or delivery system should be as similar as possible to the final nanoparticle system. Along with the necessary similarity of their basic parameters such as size and charge, the essential parameter defining the suitability of fluorescent nanoparticles as a prototype model is its stable dye retention. However, fluorescent nanosystems can be used for different purposes: (i) using the fluorescent dye as a model for a drug, which should be delivered by the nanocarrier to a target organ, (ii) developing a stable dye-nanoparticle unit for diagnostic purposes or (iii) following the distribution of the nanocarrier itself. In this respect it is of utmost importance to differentiate between the distribution patterns of the label and the carrier and to know when they exist as one entity and when they are separated.

Widely used approaches to prepare fluorescently labeled nanoparticles from prefabricated non-fluorescent polymers are the physical entrapment of a dye in nanoparticles, achieved by encapsulation during the nanoparticle formation, and the covalent binding of a dye to a core polymer [11]. Both approaches have their advantages and limitations. The encapsulation of a dye in nanoparticles is a relatively simple method based on an emulsification–solvent evaporation technique or nanoprecipitation. After solvent removal, the dye remains physically entrapped/adsorbed in the polymer matrix. One potential limitation of this technique is the dependence of adsorption or encapsulation efficiency on the physicochemical properties of both dye and polymer. Moreover, the encapsulation of a dye might change the properties of nanoparticles and the fluorescence properties of a dye, for example, by inducing fluorescence quenching or, on the contrary, enhancing emission [12]. As mentioned above, another potential drawback for nanoparticle tracking is that the dye might be prone to leakage from the nanoparticles under in vivo conditions [13]. This would eventually decrease their brightness and may create a background signal preventing proper determination of nanoparticle locations.

In contrast, the “covalent” approach enables the preparation of labeled nanoparticles with more predictable parameters that are sufficiently stable under physiological conditions [14,15]. This approach is only applicable, however, when both the polymer and the fluorophore have suitable groups for conjugation.

Stability of labeling has been evaluated previously in a number of studies (for example [13,16,17,18,19,20,21]); however, these studies employed different nanoparticles and experimental techniques for individual dyes. Based on a non-systematic pool of data, it is not easy to select the optimal experimental strategy for the preparation of the labeled particles with the desired fluorescence properties.

The objective of the present study was to study the influence of the essential parameters, such as the nanoparticle preparation procedure and the dyes’ physicochemical properties, on the label performance and properties of the resulting fluorescent nanoparticles. PLGA nanoparticles labeled with four common fluorescent dyes—DiI [22,23], coumarin 6 [24,25,26,27], rhodamine 123 [28,29,30] and Cy5.5 [31,32] were employed.

The labeled PLGA nanoparticles were compared with regard to how a dye influences their distribution, brightness (which, in turn, depends on the dye loading efficiency and quantum yield) and stability of the dye retention. Computer simulation and empirical measurements were applied for the modeling of dye leakage from the nanoparticles and the interaction of the dye molecules with lipid membranes. The stability of the encapsulated label in vitro and in vivo was then monitored using the double-labeled nanoparticles containing both the conjugated Cy5.5 and one of the encapsulated dyes. Furthermore, the nanoparticle–cell interactions were studied using confocal microscopy and in vivo neuroimaging to verify their potential value for medical applications.

## 2. Experimental

### 2.1. Materials

Poly(lactic-co-glycolic acid) (PLGA, Resomer RG 502 H, LA/GA ratio 50:50, acid terminated, Mw 7–17 kDa, η = 0.21 dL/g) was purchased from Evonik Nutrition & Care GmbH, Germany. The cyanine5.5 amine (Cy5.5 analogue) was obtained from Lumiprobe Life Science solutions (Cockeysville, MD, USA). Rhodamine 123 hydrochloride (Rh123) was purchased from Acros Organics (New Jersey, NJ, USA). Block-copolymer PLGA-PEG (PEG MW 2000 Da, PLGA MW 11,500 Da), DiI (1,1′-dioctadecyl-3,3,3′,3′-tetramethylindocarbocyanine perchlorate), coumarin 6 (Cou6), poly(vinyl alcohol) (PVA, 9 10 kDa, 80% hydrolyzed), poloxamer 188 (Kolliphor P188, BASF), N-hydroxysuccinimide (NHS), N,N-diisopropylethylamine (DIPEA), N-(3-dimethylaminopropyl)-N-ethylcarbodiimide hydrochloride (EDC), bis(2-ethylhexyl)sulfosuccinate sodium salt (AOT) and other reagents were purchased from Sigma-Aldrich (St. Louis, MO, USA). Gel filtration media Sepharose CL-2B and Sephadex G-25 were from GE Healthcare BioSciences (Uppsala, Sweden). L-α-phosphatidylcholine (egg, chicken) were purchased from Avanti Polar Lipids Inc. (Alabaster, AL, USA). All organic solvents were of analytical grade.

### 2.2. Preparation of Fluorescently Labeled Nanoparticles

PLGA nanoparticles labeled with DiI or coumarin 6 (Cou6). The PLGA nanoparticles labeled with the hydrophobic dyes DiI and Cou6 were produced by a high-pressure emulsification–solvent evaporation technique. Briefly, the polymer and the dye were dissolved in dichloromethane (DCM). The dye-to-polymer mass ratios ranged from 1:2353 to 1:39 in the case of DiI and from 1:300 to 1:25.5 in the case of Cou6. Then the organic phase was mixed with a 1% aqueous PVA solution (aqueous-to-organic phase ratio = 5:1, *v/v*), and the mixture was emulsified using a high shear homogenizer (Ultra-Turrax T18 digital, IKA, Staufen im Breisgau, Germany) followed by high-pressure homogenization at 1000 bar (Microfluidizer M-110P, Microfluidics, Newton, MA, USA) to obtain an *o/w* emulsion. After the removal of the organic solvent under vacuum, the resulting suspension was filtered through a glass-sintered filter (pore size 90–150 µm). Free dye was washed from the nanosuspension by two-step centrifugation (48,384× *g*, 5 °C, 30 min at the first step and 40 min at the second step, Avanti JXN-30, Beckman Coulter, Brea, CA, USA); each time the precipitates were resuspended in distilled water. Additionally, AOT, a bulky hydrophobic counterion, was added in the equimolar ratio to DiI during the nanoparticle preparation process (PLGA-DiI-AOT NPs) in order to decrease the self-quenching of the dye inside the nanoparticles [33]. Finally, D-mannitol was added to the suspension in an amount sufficient to produce 1% (*w/v*) concentration to act as a cryoprotectant, and the nanoparticle suspension was frozen at −70 °C and freeze-dried.

The PEG-PLGA nanoparticles labeled with coumarin 6 (PEG-PLGA-Cou6) were prepared using blends of PLGA and PLGA-PEG with varying mass ratio (PLGA:PLGA-PEG = 10:1 and PLGA:PLGA-PEG = 5:1, *w/w*). The amount of Cou6 varied from 0.3 to 3.7% (*w/w*). Homogenization was carried out as described above (15,000–20,000 psi for 1 min). A coarse nanoparticle fraction with a size >1000 nm was separated by centrifugation (15,000× *g* at 18 °C for 5 min, Avanti JXN-30, Beckman Coulter, Brea, CA, USA), and then the supernatant was additionally filtered through a 0.45 μm membrane. These conditions were sufficient to sediment the aggregated dye but not sufficient to cause significant loss of the PLGA nanoparticles in suspension. Afterwards, the nanoparticles were freeze-dried with addition of 10% (*w/v*) of mannitol. Nanoparticles were washed from the free dye as described above. Two types of the reference preparations were produced for comparison by a similar high-pressure emulsification solvent evaporation procedure: non-labeled nanoparticles (placebo PLGA nanoparticles) produced by the same technique, without adding the dye and without subsequent washing and/or coarse fraction separation, and a dye nanosuspension stabilized with PVA (polymer-free control), produced by the described techniques without adding the polymer to an organic phase and also without washing and/or fraction separation.

PLGA nanoparticles were labeled with rhodamine 123. The PLGA-Rh123 nanoparticles were produced employing a high-pressure double emulsion solvent evaporation technique as follows. An aqueous solution of Rh123 was added to the PLGA solution in DCM at a ratio of 1:48.4 (*w/w*) and emulsified using an Ultra-Turrax T18 (23,600 rpm, 1 min). Then, the obtained emulsion (*w/o*) was added to a 1% aqueous PVA solution (9–10 kDa) and passed through a multi-step homogenization process as described above. Non-encapsulated (free) dye was removed from the nanosuspension by gel filtration chromatography using a Sephadex G-25 column. The nanoparticles were freeze-dried with 1% mannitol.

Preparation of nanoparticles using PLGA conjugated with cyanine5.5 amine (PLGA-Cy5.5). The cyanine5.5 amine was covalently bound to a carboxylic terminal group of PLGA via a carbodiimide coupling reaction as described earlier [15]. For a detailed description of the procedure, see the electronic support information. The PLGA-Cy5.5 nanoparticles were produced by an *o/w* emulsion solvent evaporation technique. PLGA and PLGA-Cy5.5 (total amount 200 mg, content of pre-modified polymer 0, 50, 100, 150, 200 mg) were dissolved in 4 mL of DCM. The obtained solution was added to 20 mL of a 1% aqueous solution of PVA, mixed, and then passed through a multi-step homogenization process as described above. The organic solvent was removed using a rotary evaporator. The nanoparticles were freeze-dried with 1% mannitol.

Preparation of double-labeled PLGA nanoparticles. The double-labeled nanoparticles, i.e., the Cy5.5-PLGA nanoparticles labeled additionally with DiI, coumarin 6 or rhodamine 123 were prepared as described above using a 1:1 (*w/w*) mixture of PLGA-Cy5.5 and PLGA polymers. Thus, the PLGA-Cy5.5/DiI NPs were prepared similarly to the PLGA-DiI nanoparticles. PLGA (100 mg), PLGA-Cy5.5 (100 mg) and DiI (0.8 mg) were dissolved in 4 mL of DCM and added to a 1% aqueous solution of PVA (9–10 kDa). Cy5.5 and DiI were used in an equimolar ratio (8.6 × 10^−7^ mol). The PLGA-Cy5.5/Cou6 NPs were prepared by the same procedure as the PLGA-PEG-Cou6 nanoparticles using a blend of PLGA and PLGA-PEG polymer (5:1, *w/w*). The PLGA-Cy5.5/Rh123 nanoparticles were produced similarly to the preparation of PLGA-Rh123 nanoparticles.

### 2.3. Physicochemical Characterization

Particle size and zeta potential characterization. Average particle sizes (hydrodynamic diameter) and polydispersity indices (PDI) of the nanoparticles were measured by dynamic light scattering (DLS) using a Zetasizer Nano ZS particle size analyzer (Malvern Panalytical Ltd., Malvern, UK, equipped with He-Ne with 633 nm laser, maximum power 4 mW) at a scattering angle of 173° and a temperature of 25 °C. Samples were diluted to a concentration of 0.2 mg/mL polymer with milli-Q water. Zeta potentials were measured using the same instrument by electrophoretic light scattering (ELS) in a U-shaped disposable folded capillary cell either without dilution or diluted the same way as for the size measurements. All measurements were repeated 4 times. Sizes were evaluated immediately after preparation, before lyophilization, after lyophilization and several hours after resuspension to assess colloidal stability.

Dye content and encapsulation efficiency. The dye encapsulation efficiency (*EE_%_*) was calculated as a ratio of the dye amount entrapped in the nanoparticles to the total amount of a dye found in a sample:

(1) $$EE_{\%}=\frac{m_{total}-m_{unbound}}{m_{total}}\;\cdot \;100\%$$

The total amount of a dye was determined spectrophotometrically after dissolving samples in DMSO. Each measurement was performed in triplicate.

PLGA content. The amount of PLGA in the samples was analyzed by the amount of lactate formed after the nanoparticle destruction by hydrolysis in 1 M NaOH (37 °C, 24 h, constant shaking at 200 rpm) using capillary electrophoresis (CAPEL 105M, Lumex, St. Petersburg, Russia) as described in our previous work [15].

Scanning electron microscopy. Images of the PLGA-Cou6 nanoparticles were obtained using a scanning electronic microscope, JSM 6510LV (Jeol, Tokyo, Japan). Resolution HV mode was up to 5 nm; magnification/zoom was up to 300,000. Before the microscopical investigation, the nanoparticle samples were washed from stabilizer (PVA) and cryoprotectant (D-mannitol) by centrifugation. A drop of the resulting suspension was placed on a glass slide, dried in air and then sputter-coated with platinum for 6–30 s using an Auto Fine Coater JFC-1600 (JEOL, Tokyo, Japan). Studies were performed using the equipment of D. Mendeleev University’s Center for Collective Use equipment.

Evaluation of physical labeling stability. Stability of the nanoparticle labeling was evaluated by the assessment of the amount of the dye released from nanoparticles at chosen time points. The freeze-dried nanoparticles labeled with Rh123, Cou6 and DiI were resuspended and diluted 25-fold with the release media (1% solution of poloxamer 188 in 0.15 M PBS, pH 7.4). Then the resulting suspensions were incubated at 37 °C under continuous shaking. At chosen intervals, 1.5 mL aliquots of the suspensions were sampled, and the nanoparticles were separated by centrifugation at 48,380× *g* for 30 min at 18 °C (Avanti JXN-30, Beckman, USA). The centrifugation conditions were preliminarily optimized for the residual polymer content in the supernatant, measured by the capillary electrophoresis, as described in 2.2 (data not shown). Thus, after centrifugation of a 1.5 mL sample at 48,380× *g* at 5 °C, the PLGA content in the supernatant did not exceed 5% of the total amount. In addition, the presence of the particles was not detected by the DLS method.

The amount of the released dye in supernatants was determined spectrophotometrically upon dilution with ethanol to 50%. The experiment was performed in triplicate. The release profiles of DiI and Cou6 were additionally assessed using 1% solution of human serum albumin (HSA) in 0.15 M PBS (pH 7.4) as the release medium.

The coumarin 6 release profile from the PLGA-PEG nanoparticles was also evaluated using the dialysis technique with frequent medium replacement (Float-A-Lyzer dialysis device, cellulose ester membrane, MWCO 50 kDa, Spectrum Laboratories Inc., Rancho Dominquez, CA, USA. The release medium was a 1% solution of poloxamer 188 in 0.15 M PBS at pH 7.4. A nanosuspension of coumarin 6 was used as a non-polymeric control. At the end of the experiment, the membrane samples from the dialysis bags were dissolved in DMSO, and the amount of coumarin 6 adsorbed on the membrane was measured spectrophotometrically. The experiment was performed in triplicate.

Fluorescence measurements. Fluorescence quantum yields (QY) were determined using an adaptation of the comparative method described by Rhys Williams et al. [34] and the measurement conditions recommended by IUPAC [35]. The method is based on measurements of the fluorescence spectrum of the sample and comparison of its integrated intensity with the same quantity of a reference system, i.e., a certain standard with a known absolute QY and similar maxima of absorption and emission.

The general procedure was as follows. A series of standard solutions with varied concentrations and a series of suspensions of varied dilutions were prepared. Standards were chosen, when possible, based on the data of Brouwer [35]. The following standard solutions were used: rhodamine 6G solution in water for the PLGA-DiI nanoparticles, coumarin 153 solution in ethanol for the PLGA-Cou6 nanoparticles and Rh123 in ethanol for PLGA-Rh123, Cy5.5 in 96% ethanol for PLGA-Cy5.5 nanoparticles. Then, absorbance was measured for each sample at the relevant excitation wavelengths (488 nm for PLGA-DiI NPs, 450 nm for PLGA-Cou6 NPs, 480 nm for PLGA-Rh123 NPs, 684 nm for PLGA-Cy5.5 NPs). Absorbance values used were ≤0.25 in order to minimize the reabsorption effects. The fluorescence spectra were recorded for the same suspensions and solutions using the aforementioned wavelengths for excitation (RF-6000, Shimadzu, Japan). Optical path length was 10 mm, and slit widths for excitation and emission were held constant (5 nm). Integrated intensities were obtained for each sample. To calculate the relative quantum yields (*QY_R_*), the intensities were plotted against absorbances of the corresponding samples. The slopes of the acquired graphs were used in the following equation:

(2) $$QY_{r}=QY_{st}\cdot \frac{tg\alpha_{x}}{tg\alpha_{st}}\cdot \frac{n_{x}^{2}}{n_{st}^{2}}$$

where *QY_st_* is the absolute quantum yield of the reference (0.90 for rhodamine 6G [36], 0.20 for cyanine5.5 amine (manufacturer’s data) and 0.53 ± 0.02 for coumarin 153 [37]); *tgα_x_* and *tgα_st_* are the slopes of the graphs of nanoparticles and standards, respectively; *n_x_* and *n_st_* are the refractive indexes of solvents (1.3333 for water and 1.3617 for ethanol). The specific brightness of fluorescence was calculated accordingly as:

(3) Specific brightness=ε·QY·n

where *ε* is the molar extinction coefficient of the dye loaded in nanoparticles calculated for a given sample calculated using the Beer–Lambert law, l/mol·cm; *QY* is the quantum yield, dimensionless (fraction of 1); *n* is the amount of substance of fluorophore molecules per mg of PLGA, mol/mg.

For nanoparticles labeled with both DiI and Cy5.5, FRET efficiency was calculated as described in the work of Majoul et al. [38] (See Supplementary Materials.)

Dye partitioning in liposomes. The liposomes were prepared by lipid membrane hydration. Briefly, L-α-phosphatidylcholine (20.4 mg) and cholesterol (4.8 mg) were dissolved in 5 mL of chloroform. The organic solvent was removed under vacuum at 40 °C until the lipid film was formed. Further, 20 mL of 0.01 M PBS (pH 7.4) was added, and the mixture was sonicated in an ice bath for 2 min at 100% power (Sonopuls HD2070, Bandelin, Berlin, Germany). The resulting suspension was filtered through a glass porous filter (pore size 100–160 μm).

For evaluation of the dye leakage, the liposomes (2–5 mL) were mixed with the fluorescent PLGA nanoparticles resuspended previously in 1 mL of 20% sucrose solution in 0.01 M PBS (pH 7.4). The control samples were prepared without the addition of liposomes. All samples were incubated for 1 h at 37 °C at constant shaking (200 rpm). Then the nanoparticles and the lipid fraction were separated by centrifugation (5 °C, 30 min, 15,000× *g*). The supernatants containing liposomes were removed, and the precipitates (nanoparticles) were resuspended, frozen and freeze-dried. The amount of the dye in precipitates was measured spectrophotometrically after their dissolution in DMSO, as described above. The total amount of the dye transferred to liposomes was calculated as the difference between the concentrations in control and in the sample (*c_contr_* − *c_lipo_*). The amount of a free dye was taken into consideration through encapsulation efficiency. To calculate the fraction of a dye transferred to liposomes (*T*), the following equation was applied:

(4) $$T=\frac{\left(C_{contr}-C_{lipo}\right)\cdot EE}{C_{contr}}\cdot 100\%,$$

where *c_contr_* is the total amount of the dye, measured in precipitate of the control sample; *c_lipo_* is the residual amount of the dye in the nanoparticles after incubation with liposomes; *EE* is the encapsulation efficiency.

In this experiment, two types of DiI samples were used: in one case, the free dye was removed by centrifugation (5 °C, 48,380× *g*, 30 min), in another by means of GPC (Sepharose CL-2B), followed by centrifugation as described above.

Differential scanning calorimetry. Glass transition temperature (T_g_) was measured for the Cou6-PLGA and DiI-PLGA nanoparticles. Non-loaded PLGA nanoparticles and bulk polymer were used as control. The samples of Cou6-PLGA and DiI-PLGA nanoparticles used for the DSC analysis were prepared as described above at the dye/polymer weight ratio of 1:20 and were not subjected to washing procedure. The calorimetric measurements were performed using differential scanning calorimeters TA-4000 equipped with a DSC-30 heating cell and DSC823e (Mettler Toledo GmbH, Greifensee, Switzerland) at a heating rate of 10 °C/min under argon. The samples were measured in aluminum pans. The temperature range was −20 °C to 100 °C. A blank aluminum pan was used as reference. All glass transition temperatures (T_g_) were reported as the onset of the transition.

Modeling of dye interaction with lipid membranes. The model lipid membranes consisted of either dipalmitoylphosphatidylcholine (DPPC) or dimyristoylphosphatidylcholine (DMPC) bilayers. The analysis of the dyes’ conformational isomers (conformers) -- their structure and stability—was carried out using a composite hierarchical approach. Molecular mechanics (MM) was employed to perform the initial screening of conformers with the OPLS3e force field [39] as implemented in the conformer advanced search script [40]. At this step, for each type of the dye molecules, 100 lowest-energy conformers were selected, the structures and energetics of which were further refined in semi-empirical electronic structure calculations at the PM6-D3H4 level [41] using the MOPAC2016 program package (MOPAC2016, supported by J.P. Stewart, Stewart Computational Chemistry, Colorado Springs, CO, USA). Next, the results were clustered with RMSD and energy to determine the most stable conformers, which were further used for the DFT calculations with the solvation model. Finally, the COSMO-RS approach was used to figure out the Boltzmann distribution of conformers for each dye, which allows us to find solubilities and partition coefficients more accurately. DFT calculations were carried out using the Turbomole program package (TURBOMOLE V7.2 2017, development of University of Karlsruhe and Forschungszentrum Karlsruhe GmbH) at the BP86/TZVPD level of theory [42,43,44,45] as a recommended procedure for COSMO-RS calculations [46]. The solubility of dyes and their partition coefficients in bi-phase systems were then computed using a hybrid COSMO-RS solvation model [46,47] with CosmoTherm 16 program [48]. Polymers (PLA and PLGA) were simulated with CosmoTherm 16 program according to the recommended approach [49].

### 2.4. Biological Studies

In vitro visualization. The 4T1 murine breast carcinoma cells were purchased from the American Type Culture Collection (ATCC, Manassas, VA, USA). The cells were cultured at a seeding density of around 50,000/cm^2^ in Ham’s F-12K medium (2.5% fetal bovine serum, 15% horse serum and 1% penicillin) and DMEM (10% newborn calf serum, 100 U/mL penicillin and 100 mg/mL streptomycin), respectively, at 37 °C in 5% CO_2_ for 24 h to allow cell attachment. Before use, nanoparticles were additionally coated with a surfactant, which was accomplished by resuspending the samples in a 1% poloxamer 188 aqueous solution and incubating them for 30 min. Then, nanoparticles at a final concentration of 100 µg/mL were added to the fresh medium, and the cells were incubated from 0 to 20 min. After the incubation, the cells were gently washed three times with PBS and further incubated with 2.5 µg/mL of Hoechst, 50 nM/mL of Lysotracker Green DND26 for 20 min and then again washed three times with PBS. The cells were observed using a confocal laser scanning microscope (inverted multiphoton confocal microscope Nikon A1 MP, Nikon Instruments Inc., Tokyo, Japan) for 20 min. FRET imaging was applied to the double-stained nanoparticles after their incubation with the cells for 20 min.

In vivo confocal neuroimaging (ICON). All procedures were performed with ethical approval according to the requirement of the German National Act on the use of experimental animals (Ethic committee Referat Verbraucherschutz, Veterinärangelegenheiten; Landesverwaltungsamt Sachsen-Anhalt, Halle, approval code: 203.6.3-42502-2-1469 UniMD_G/09.07.2018). Adult male Lister hooded rats (Crl: LIS strain; Charles River) were housed on a 12 h light/12 h dark cycle under standard environment conditions at ambient temperature of 22 °C at 50–60% humidity. Animals had access to food and water ad libitum, except on the day before the induction of narcosis, when food was removed. Animals were kept at least one week for adaption in group cages and were handled before starting the experiment to reduce stress.

Cy5.5-PLGA-Cou6 NPs for ICON imaging were dispersed in 0.5 mL of a sterile 1% poloxamer 188 solution in deionized distilled water (dd water). Fluorescent signals of Cy5.5-PLGA-Cou6 NPs were observed as described in previous studies [50,51]. Briefly, the animals were anesthetized by an intraperitoneal injection of ketamine (75 mg/kg) and medetomidine (0.5 mg/kg) and kept on a heating pad. A cannula was inserted into the tail vein for application of the NP solution. The irises of the eyes of anaesthetized rats were dilated with neosynephrine-POS 5%. A contact lens was adjusted onto the cornea and immersed in Vidisic eye gel. Then, the rats were fixed under a confocal scanning microscope with the eye positioned in working distance underneath the objective of a Zeiss LSM 880 microscope, and in vivo confocal neuroimaging (ICON) of the retina was performed. The images were captured at time point 0 min (baseline before NP injection) and post-injection at 40 min (excitation/emission wavelengths used for coumarin 6 and Cy5.5 were 488/502 nm and 633/708 nm, respectively). The rats were kept on the heating pad throughout the in vivo imaging process. All the images were obtained with the same resolution (5× magnification and same image size).

Statistical analysis. Student’s *t*-test and ANOVA on ranks were used to investigate the differences statistically. The differences were considered significant at values of *p* < 0.05. All values were expressed as mean ± SD.

## 3. Results & Discussion

The dyes investigated in this study are widely used to visualize nanoparticles in vitro and in vivo. However, data regarding their properties, especially the values of partition coefficients characterizing the distribution of the dyes in various media, differ from one source to another or are absent in the literature. This is mostly due to the variety of software used for calculation based on various algorithms or, in the case of experimental values, it is because these dyes are so poorly soluble that it is hard to obtain a reliable value. Nevertheless, one can with certainty claim that the dyes’ hydrophobicity increases in the following order: Rh123—Cou6—DiI and that DiI is significantly more hydrophobic than Cou6 and Rh123 (Table S1). Those properties (namely, poor solubility in aqueous media and hydrophobicity) in many cases determine the choice of dye used in the visualization of nanoparticles. Researchers believe that these properties allow the dyes to be easily encapsulated into nanoparticles and to remain in the nanoparticles during in vitro/in vivo experiments. Moreover, for the same reasons, coumarin 6 is used not only as a bright and convenient label for the visualization of various nanocarriers [25,26] but is also considered a suitable model of a hydrophobic drug [52,53]. In order to further investigate the acceptability and reliability of the aforementioned dyes as labels for nanoparticles, we have conducted this study concerning various aspects that are important for fluorescent nanoparticles: colloidal stability, brightness, dye retention, interaction with biological media, etc.

### 3.1. Preparation and Basic Properties of the Labeled Nanoparticles

Due to their hydrophobic nature, DiI and coumarin 6 are insoluble in water but readily dissolve in dichloromethane (DCM), as does the polymer PLGA; therefore, these nanoparticles could be obtained by a simple (*o/w*) emulsification–solvent evaporation method. Similarly, for the polymer conjugated with Cy5.5, the same technique was employed. Rhodamine 123 hydrochloride is more soluble in water than in DCM; therefore, in this case the PLGA-Rh123 nanoparticles were obtained by a double (*w/o/w*) emulsification technique. Initial dye-to-polymer ratios were chosen based on preliminary experiments so that they could enable the visualization of the nanoparticles in the in vitro and in vivo experiments.

In general, the encapsulation of both DiI and rhodamine 123 was efficient and did not produce any considerable changes in the basic parameters of the nanoparticles: these nanoparticles had mean diameters of ~100 nm with relatively low PDI (<0.2) and negative zeta potentials (Table S2). In the case of the nanoparticles labeled with DiI, the final dye-to-polymer ratio varied in the range of 0.2–12.5 µg/mg (initial loadings were in the range of 0.4–25.6 µg/mg) due to losses during the preparation and washing procedures. In this range, no influence of the dye-to-polymer ratio on the encapsulation efficiency of DiI was observed; the average value was around 70%.

Resomer RG 502 H used in this study as a core polymer has a terminal carboxylic end group that enabled the labeling of the nanoparticles by its conjugation with the reactive amine derivative of Cy5.5. Labeling of PLGA with cyanine5.5 amine was performed using a carbodiimide method. The absence of fluorescence in the aqueous phase upon purification of the conjugate by washing signified the quantitative conjugation. The absence of unbound dye in the final preparation was evidenced by TLC. TLC also demonstrated that the label was stable after the nanoparticle preparation.

The PLGA-Cy5.5 nanoparticles were produced using different ratios of pre-modified PLGA-Cy5.5 polymer and the original polymer while keeping the total polymer amount constant. The presence of the modified polymer did not exert any influence on the nanoparticles’ basic parameters: the average size of all PLGA-Cy5.5 nanoparticles was about 120 nm (PDI < 0.2), with a zeta potential of around −22 mV (Table S2).

In the double-labeled PLGA nanoparticles, containing one of the aforementioned dyes and covalently attached Cy5.5, the latter served as a marker, allowing for a more accurate analysis of the localization of both the particles and the physically entrapped dye, whether it is still associated with the nanoparticles or has leaked out. This approach is often a much-needed tool because otherwise it is hard to recognize the difference between a signal generated by a nanoparticle and a signal of the released dye, in part due to resolution limitations. The data obtained in this study indicate that double labeling represents a useful tool to verify the stability of the fluorescent dye incorporated into the nanoparticles (see below).

**Figure 1:**
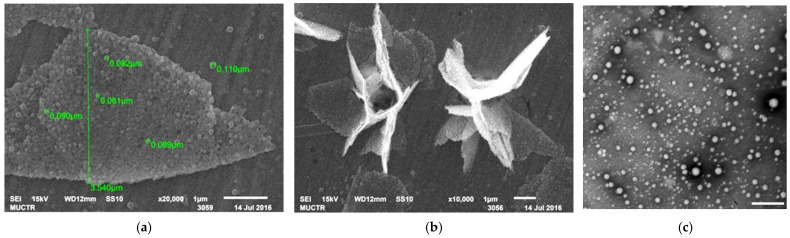
(**a**): SEM image of PLGA-Cou6 nanoparticles (non-PEGylated); (**b**): nanoparticles clusters (broader view); scale bar 1 μm; (**c**): PLGA-PEG-Cou6 nanoparticles (scale bar 500 nm).

Among the nanoparticles prepared and investigated in this study, the PLGA-Cou6 nanoparticles labeled with coumarin 6 exhibited a distinctive behavior. These nanoparticles were characterized by a high encapsulation efficiency of 93–99% with a dye content of 12–17 µg/mg PLGA at the initial dye-to-polymer ratio of 1:170 (*w/w*). Interestingly, despite the higher solubility of coumarin 6 compared to DiI (Table S1) and its lower hydrophobicity (calculated logP 5.0 and 10.3, respectively see Section 3.5), the PLGA-Cou6 nanoparticles had a higher polydispersity index (PDI >0.2) and a less negative surface charge (−8.5 ± 0.6 mV) than the PLGA-DiI nanoparticles, which obviously was due to the dye adsorption on the nanoparticle surface leading to their increased hydrophobicity and the formation of aggregates. A tendency towards agglomeration with the formation of unusual clusters was also observed in the SEM images of these nanoparticles (Figure 1a,b).

This unwanted phenomenon could be avoided by using a mixture of PLGA and PEG-PLGA in nanoparticle preparation (Figure 1c), a technique frequently used to increase colloidal stability [54]. The hydrophilic brush on the nanoparticle surface created by the PEGylated polymer could considerably decrease agglomeration, and, accordingly, lead to the formation of smaller Cou6-labeled nanoparticles with lower polydispersity (see Supplementary Materials). However, the presence of the PEG moiety also changed the surface properties of these nanoparticles compared to the plain PLGA nanoparticles. Overall, it is hard to consider coumarin 6 a “neutral” fluorescent label. Therefore, it is advisable to use Cou6 in small concentrations as a fluorescent label; moreover, the nanoparticles should be thoroughly washed from the free dye, however, the washing procedure might not be possible if the target therapeutic nanoparticles preparation procedure does not imply it. At the same time, the encapsulation of DiI and Rh123 did not lead to significant changes in the nanoparticles’ physicochemical properties.

The double-stained nanoparticles were obtained to get insight into the process of dye leakage in biological media. The physicochemical parameters of the double-labeled nanoparticles were similar to those of the respective single-labeled and non-labeled nanoparticles (Table S5). For the PLGA-Cy5.5/DiI nanoparticles, the encapsulation efficiency of DiI was on average higher compared to the PLGA-DiI nanoparticles.

### 3.2. Stability of the Nanoparticle Labeling

After having determined the optimal dye-to-polymer ratio, it is equally important to evaluate the stability of the fluorescent label in nanoparticles under conditions simulating the biological environment (lipophilic or hydrophilic or with different pH levels). However, it is challenging to mimic the physiological conditions in test tube conditions for release investigation, especially for hydrophobic substances [55]. As highlighted by Simonsson et al. [56], the classical methods based on employment of water or buffer as the release medium will not mimic the release in an in vivo environment, where multiple compartments are present. Various methods are used to evaluate the transfer of dye from nanocarriers to the cells: the incubation of the nanoparticles with cells at 4 °C to avoid the active transport [17]; the use of lipid acceptor compartments mimicking the cell membrane, such as liposomes, oils and other lipids [16,57]; and the application of the FRET phenomenon [20]. In this study, we applied three complementary approaches: common in vitro dye release studies in aqueous media, incubation with liposomes and computer simulations (prediction of dye interaction with lipid bilayer).

The stability of nanoparticle labeling was evaluated by an assessment of the dye fraction released after 2 h and 24 h of incubation (Table 1). The in vitro release of the encapsulated agents from the nanoparticles is generally analyzed in aqueous media simulating body fluids, such as, for example, phosphate buffered saline (PBS, pH 7.4). However, among the dyes used for encapsulation in this study, only rhodamine 123 was slightly soluble in PBS, others were practically insoluble. To prevent precipitation of the dyes during the continuous experiment, a 1% solution of poloxamer 188 in 0.15 M PBS (pH 7.4) was used as a release medium. However, even in the presence of the surfactant, the reliable results were obtained only within 2 h of incubation. At later time points, the dyes’ precipitation interfered with the accuracy of measurements. Therefore, considering that studies on nanoparticle trafficking are mostly performed using plasma-containing media (i.e., in cell culture experiments or upon administration of the particles in the bloodstream), the stability of label retention was also evaluated in the presence of human serum albumin (HSA, 1% solution in PBS) as the most abundant plasma protein.

All dyes demonstrated a different behavior in the release studies. Rhodamine 123 was quickly released from the nanoparticles. For PEG-PLGA-Cou6 nanoparticles, the release rate depended by the medium: Cou6 was released slowly in a 1% poloxamer 188 solution but much faster in a 1% HSA solution, as HSA enhanced its solubilization. In contrast, the initial release of DiI was much slower in an HSA solution compared to the poloxamer 188 solution. However, after 24 h incubation in the presence of HSA, the free dye concentration substantially increased (Table 1).

In the protein-free medium, DiI labeling was the most stable: approximately 10% of the dye was released from the PLGA-DiI nanoparticles after 2 h incubation. The PEG-PLGA-Cou6, nanoparticles released 17% of the dye content in these conditions. In contrast, the PLGA-Rho123 demonstrated a considerable burst-effect: 57% was released during the first 2 h (more likely desorbed from the surface); however, after this period the release was slow and, after 24 h, about 45% of the dye remained bound to the nanoparticles. There was no release of the conjugated Cy5.5 during 24 h incubation and up to 7 days.

Importantly, while the retention of DiI and coumarin 6 was seemingly sufficient in the protein-free medium, in the presence of HSA, the release rates of both dyes were enhanced considerably. This phenomenon is most probably explained by the known ability of HSA to form complexes with lipophilic molecules, which contributes to their more effective transfer from the nanoparticles into the aqueous medium. However, while the released fraction of coumarin 6 after 2 h of incubation was above 40%, only 1% of DiI was released from the nanoparticles at this time. Thus, in terms of label stability, the PLGA-DiI nanoparticles appear to be a reliable system for short-term imaging experiments in vitro and in vivo.

Both DiI and coumarin 6 are known for their ability to stain cell membranes, which is in line with their lipophilicity. Moreover, the rapid redistribution of coumarin 6 from nanocarriers into cell membranes was observed in the in vitro experiments [58,59]. The rate of coumarin 6 redistribution also depended on the type of the carrier and was higher in the case of the liposomes compared to the solid lipid nanoparticles. The role of the nanocarrier in the dye partitioning into the cell membranes probably explains the different results obtained for the lipid nanocapsules and PEG-polystyrene nanoparticles labeled with DiI: whereas the lipid-based carrier enabled a stable retention of the dye in the presence of cell membranes [56], in the case of polymeric nanoparticles, the release rate was strongly facilitated [60]

The use of dialysis for the separation of the nanoparticles from free coumarin 6 was unsuccessful due to the dye adsorption on the membrane (6.3%) and its slow diffusion through the membrane (after 96 h, the amount of the released dye in the medium was <1.6%). Similar results were obtained for the non-polymeric formulation of Cou6 (suspension). Hence, in the case of coumarin 6, the use of the dialysis technique may lead to the misinterpretation of the dye release rate from the nanoparticles.

No leakage of the covalently linked dye (Cy5.5) was observed for at least 24 h in various media (water, poloxamer 188 or HSA solution) (data not shown). Interestingly, labeling with DiI appeared to be more stable in the double-labeled PLGA-Cy5.5/DiI nanoparticles: after two weeks of incubation, 27.5% of DiI was released from the PLGA-DiI nanoparticles, whereas, in the case of the PLGA-Cy5.5/DiI nanoparticles, only 13.8% of the dye was released during this time (discussed below).

Covalently bound Cy5.5 label was not found in liposomes indicating that, under the experimental conditions, all of the dye remained associated with the nanoparticles. This result confirms that covalent conjugation of the dye with the core polymer is one of the most reliable ways to provide a stable staining of the nanoparticles. As shown in our previous study, the doxorubicin-loaded PLGA nanoparticles labeled with covalently bound Cy5.5; this approach yields accurate results on the nanoparticle uptake and localization within cells [15].

An interesting phenomenon of a higher encapsulation efficiency and slower release rate in the case of the double-stained PLGA-DiI/Cy5.5 nanoparticles was probably caused by the π-stacking between the molecules of these dyes, which contributed to the retention of DiI in the polymeric matrix due to electrostatic interaction [61]. A similar approach is described for polymeric micelles loaded with doxorubicin: a part of the total amount of doxorubicin was covalently bound to the carboxylic groups of poly(aspartate) micelles, whereas another part was physically loaded into the micelles. This approach enabled the better retention of doxorubicin in the polymeric micelles due to π-π interactions between free and covalently bound doxorubicin molecules [62]

### 3.3. Fluorescent Properties of the Labeled Nanoparticles

A very important characteristic of a fluorescent label is its brightness, which determines the sensitivity of its detection. In the case of suspension of polymeric nanoparticles labeled with a dye, the difficulty of brightness evaluation lies in the contribution of light scattering, which may interfere with the accuracy of measurements. In addition, the polymer environment and the dye–dye interaction, depending on the dye-to-polymer concentration, can influence the fluorescent properties (QY, *ε*) of dye molecules inside the nanoparticles. These factors complicate comparison of the properties of fluorescent nanoparticles in suspension with a solution of the dye itself. In this study, the specific brightness values were determined, because this parameter is very convenient for comparing different types of fluorescent particles [63]

#### 3.3.1. PLGA Nanoparticles with a Single Label

**Figure 2:**
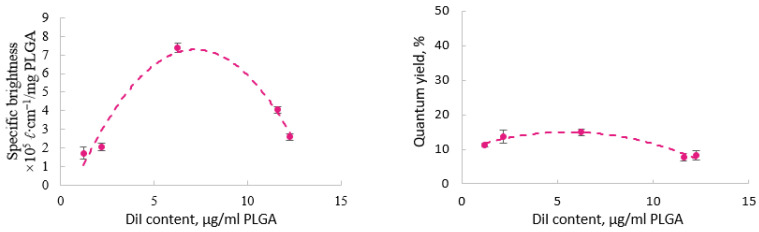
Influence of DiI content in PLGA nanoparticles on their specific brightness (**left**) and on quantum yield (λ_EX_ 488 nm) (**right**).

The extent to which a dye-to-polymer mass ratio influenced the optical properties (quantum yield and brightness) of nanoparticles varied. In the case of the PLGA-DiI nanoparticles, the highest QY (14.91 ± 0.16%) was observed for the nanoparticles with a DiI content of 6.3 μg/mg PLGA (0.63 wt.%). (Figure 2). These particles also exhibited the maximal brightness (7.3 × 10^−5^ ℓ cm^−1^/mg PLGA). As a result, the DiI content in the nanoparticles that provided the maximal brightness was found to be ~5 μg/mg PLGA.

A further increase of the dye concentration in the nanoparticles led to a decrease in the quantum yield and brightness, which is most likely associated with aggregation-caused quenching (ACQ). Interestingly, in contrast to our expectations, a bulky counter-ion, bis(2-ethylhexyl) sulfosuccinate (AOT) that was added in order to prevent fluorescence self-quenching of DiI (PLGA-DiI-AOT nanoparticles) led to a two-fold decrease in the fluorescence QY (from 13.72 ± 0.16% to 6.58 ± 0.03%); at the same time, their brightness increased by about 1.5 times (from 2.06 × 10^−5^ to 3.14 × 10^−5^ ℓ cm^−1^/mg PLGA) due to the increased number of fluorophore molecules in the PLGA core. Possibly this decrease of the fluorescence QY is associated with the formation of a non-luminescent ion pair DiI-AOT. Moreover, the zeta potential of the PLGA-AOT-DiI nanoparticles was almost neutral, thus being substantially different from the negatively charged non-labeled and DiI-labeled nanoparticles prepared without AOT (−0.2 ± 0.2 mV versus −40.7 ± 2.1 mV, respectively).

**Figure 3:**
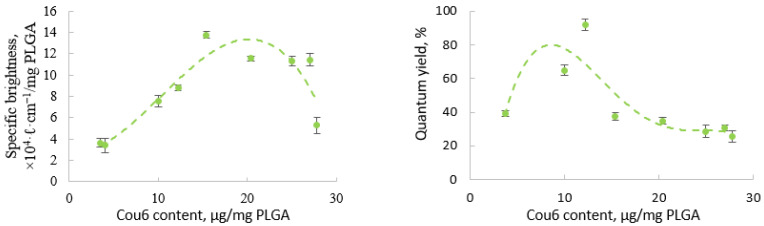
Influence of Cou6 content in PEG-PLGA nanoparticles on their specific brightness per mg of PLGA (**left**) and on quantum yield (**right**) (λ_EX_ 450 nm).

The optical properties of the PEG-PLGA-Cou6 nanoparticles also depended on the dye content (thoroughly washed nanoparticles were used for the measurements). The maximal QY (91.9 ± 0.2%) was observed for the nanoparticles containing 12.2 μg of dye per 1 mg of PLGA (Figure 3). These nanoparticles also demonstrated the highest specific brightness, ranging from 3.39 × 10^−4^ to 13.37 × 10^−4^ ℓ cm^−1^/mg PLGA A, and the number of fluorophore molecules per mg PLGA was ten times higher than in the PLGA-DiI NPs. At higher dye concentrations, the QY of the fluorescence decreased due to concentration quenching.

The Rh123-labeled nanoparticles had a QY of 46.8% and specific brightness of 2.02 × 10^−4^ ℓ cm^−1^/mg PLGA that was 3-fold higher than that of the brightest PLGA-DiI NPs but lower compared to the PLGA-PEG-Cou6 nanoparticles. No further optimization of the Rh123-labeled nanoparticles was performed because of the fast release of this dye.

**Figure 4:**
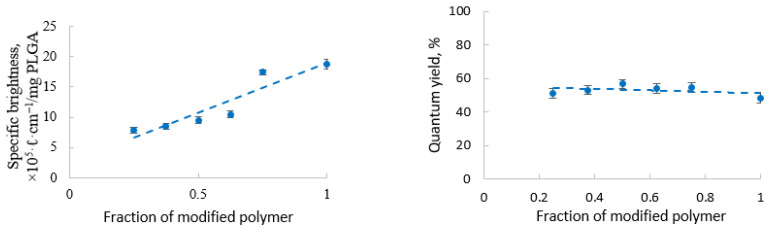
Influence of the PLGA-Cy5.5 content in nanoparticles on brightness (**left**) and quantum yield (**right**).

Quantum yield of Cy5.5 in the PLGA-Cy5.5 nanoparticles was significantly higher than that of a free dye in ethanolic solution (~50% versus 20%, respectively), which is probably due to the known increase in the fluorescence properties of indocyanine dyes in a viscous microenvironment [64,65]. In this case, no significant influence of the fluorophore amount on the quantum yield was observed in the range from 0.41 μg/mg to 1.66 μg/mg PLGA: in all cases, QY was about 50%. Brightness, however, increased along with the PLGA-Cy5.5 content in the nanoparticles (Figure 4).

**Figure 5:**
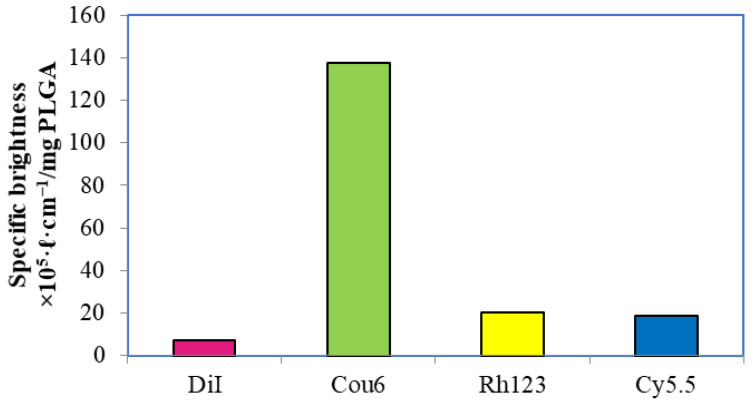
Maximal specific brightness values for the PLGA nanoparticles with a single label.

As shown in Figure 5, the PEG-PLGA-Cou6 nanoparticles were by far the brightest among the single-labeled nanoparticles.

#### 3.3.2. PLGA Nanoparticles with a Double Label

Emission spectra of the dyes, which were used in pairs, do not overlap significantly, and all dyes retained their characteristic emission peaks (Supplementary Materials, Figures S1–S4). The absence of overlap is advantageous for the signal discrimination in in vitro and in vivo experiments. On the other hand, the overlap between emission and excitation spectra of two dyes indicates that the pair will display Förster resonance energy transfer (FRET), where the magnitude of the effect depends on the area of overlap. Among the pairs used, such overlap was the greatest for Cy5.5 and DiI, followed by the Cy5.5–Rh123 and Cou6–Cy5.5 pairs.

Accordingly, the most considerable FRET phenomenon was observed for the DiI/Cy5.5 pair (not reported previously). The efficiency of FRET calculated according to Equation (4), using the quantum yields for DiI in the double-labeled (Cy5.5-PLGA-DiI) and DiI-labeled (PLGA-DiI) nanoparticles of 11.3% and 32.5%, respectively, was 65.2%, which correlated with the molar ratio of the dyes in the nanoparticles. FRET was also observed in the case of DiI and Rh123. However, the efficiency of Rh123/Cy5.5 energy transfer was not sufficient for application in the in vitro and in vivo experiments.

A rather considerable overlap between the fluorescence spectrum of DiI and absorbance spectrum of Cy5.5 offers the possibility of using FRET as an additional method of monitoring nanoparticle stability. The fact that an intensive FRET signal was observed in the cells after 1 h of incubation confirms that no leakage occurs at that time, which correlates with the results of the release studies.

### 3.4. Evaluation of the Dyes’ Partitioning in Liposomes

Both DiI and Cou6 exhibit affinity to plasma membranes and other lipid structures [66,67], which is in line with their lipophilicity. Thus, rapid redistribution of coumarin 6 from nanocarriers into cell membranes was observed in the in vitro experiments [58,68]. The rate of coumarin 6 redistribution also depended on the type of the carrier and was higher in the case of the liposomes as compared to the solid lipid nanoparticles. The role of the nanocarrier in the dye partitioning into the cell membranes probably explains also the different results obtained for the lipid nanocapsules and PEG-polystyrene nanoparticles labeled with DiI in the presence of cell membranes: whereas the lipid-based carrier enabled a stable retention of the dye [56], in the case of the polymeric nanoparticles, the release rate was strongly facilitated [60].

Therefore, for the proper evaluation of nanoparticle biodistribution, it is important to evaluate the affinity of the encapsulated dyes to the cell membranes. Liposomes can act as the model cell membranes mimicking the arrangement of the lipids in natural cell membranes and therefore are frequently used for this purpose. For this purpose, in order to evaluate the role that lipid components may play in the dye leakage from the nanoparticles, the dye retention was tested in the medium containing liposomes. The Z-average diameter and PDI of the liposomes employed in the present study were 307 ± 21 nm and 0.139 ± 0.010, respectively. The dye-loaded and washed nanoparticles were incubated with liposomes for 1 h; then, the liposomes and nanoparticles were separated by centrifugation in 20% sucrose, followed by analysis of both fractions. In the case of properly washed PLGA-DiI and PLGA-Cy5.5 nanoparticles, no dye transfer to the lipid layer was observed, whereas for the non-washed PLGA-PEG-Cou6 or PLGA-DiI nanoparticles, the fraction of a dye transitioned into liposomes was about 62% and 38%, respectively (Table 2).

### 3.5. Modeling of Dyes’ Interaction with Model Lipid Membranes

**Figure 6:**
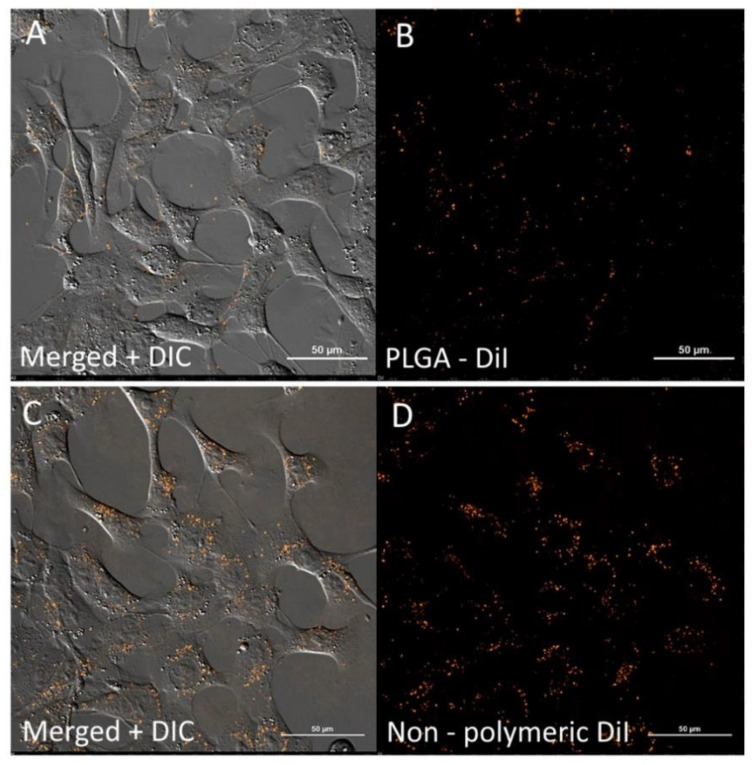
Laser scanning confocal microscopy of 4T1 cells after 40 min of incubation with DiI nanosuspension: PLGA-DiI NPs (**A**,**B**) and non-polymeric DiI formulation, stabilized with 1% PVA) (**C**,**D**). (**A**,**C**)—merged image and differential interference contrast (DIC); (**B**)—PLGA-DiI fluorescence; D—non-polymeric DiI fluorescence. Scale bar 50 μm.

Thorough the removal of non-encapsulated DiI (preferably by GPC, followed by centrifugation) is important for a reliable interpretation of the imaging results in vitro and in vivo, since dye molecules that are loosely associated with nanoparticles easily distribute into the cell membranes, and, therefore, may create artifacts upon visualization. This pattern of the DiI behavior was further confirmed by computer simulation data (DFT). DiI is the most hydrophobic of the studied dyes and is practically insoluble in water (Table 3). It appears that the affinity of DiI to the model lipid membrane (DPPC/DMPC) is much higher than its affinity to the polymer. This relatively low affinity to the polymer explains the appearance of a considerable fraction of the dye adsorbed onto the surface of the nanoparticles, which is due to its tendency to form its own phase at the boundary of the emulsion droplet during the particle preparation process. In an aqueous medium, the molecule of that dye will “prefer” the lipid membrane to the polymeric matrix, given that there is a choice. In the absence of direct contact with the lipid layer, the probability that the dye molecule will transit from the nanoparticle surface into water and then to the membrane is negligible, since the solubility of DiI is very low. However, when the PLGA-DiI nanoparticles come in contact with the lipid membrane, the partition of the surface-adsorbed DiI into this membrane is a quick and efficient event, as was observed in the experiment wherein the PLGA-DiI nanoparticles were incubated with the liposomes. Indeed, in the case of the insufficiently washed PLGA-DiI nanoparticles, ~38% of DiI leaked into the liposomes. The results concerning the DiI affinity to the cell membrane in various publications are rather different [17,20,56]. Although lipophilic DiI is widely used as a cellular membrane tracer in biological studies [69], we observed that both the PLGA-DiI nanoparticles and non-polymeric DiI nanosuspension were internalized into the cells and distributed within the cytoplasm (Figure 6). No membrane staining was observed. Moreover, confocal images of the double-stained PLGA-Cy5.5/DiI nanoparticles confirmed the colocalization of DiI and Cy5.5 fluorescence signals. The stabilization of the dye in aqueous media could possibly prevent its diffusion into the cellular membrane. Indeed, according to several protocols recommended for staining of cellular membranes and for retrograde and anterograde neuronal labeling, the lipophilic dyes (DiI, DiO, DiD) should be first dissolved in DMSO and then applied to the samples in ethanolic solution [70].

Computer simulation is another useful approach for predicting the behavior of a fluorescent dye loaded in nanoparticles in vitro. To estimate the possibility for each of the dyes to exit the nanoparticles into the aqueous medium and to enter a model lipid membrane composed of either dipalmitoylphosphatidylcholine (DPPC) or dimyristoylphosphatidylcholine (DMPC), we have employed the DFT simulations in the frames of a COSMO-RS model. This model allows the calculation of the chemical potentials of substances depending on the temperature, pressure, concentrations and composition of the mixture. Thus, it is suitable for the computation of various physicochemical properties. This approach is a well-known method used for the evaluation of the solubilities and partition coefficients of small organic molecules in polymers [49,71]. Also, the chosen computational approach is quite sensitive for predicting the partition coefficients of the drug-like molecules, which was demonstrated in the blinded test challenges SAMPLE5 [72] and SAMPLE6 [73].

To investigate the activity of the studied dyes in complex multicomponent media, phase distributions for each compound in all examined systems were calculated. For each dye, only the most common conformers in vacuum, liquids and DPPC or DMPC bilayers were obtained. The final conformers of both DPPC and DMPC molecules were chosen as the components of a membrane lipid bilayer (see section of Supplementary Data “Modeling of dye interaction with lipid membranes” and Supplementary Table S6). Since nanoparticle formation occurs at ambient temperature, the computations were conducted at 25 °C (298.15 K). At this temperature, the DMPC bilayer exists in the liquid crystalline state (gel-to-liquid crystalline transition temperature 24 °C), whereas the DPPC bilayer exists in the gel state (gel-to-liquid crystalline transition temperature 41 °C) [74].

According to the obtained data, rhodamine 123 hydrochloride is the only dye that dissolves well both in water and in hydrophobic media: log P_DPPC/WAT_ and log P_DMPC/WAT_ is equal to −0.58, and the calculated log P_OCT/WAT_ value is equal to 0.04. Both DiI and Cou6 are soluble in hydrophobic liquids only. For coumarin 6, the log P_OCT/WAT_ value is equal to 5 and both log P_DPPC/WAT_ and log P_DMPC/WAT_ values are equal to 5.2. Interestingly, the difference in the lipid bilayer composition and phase state did not affect the dyes’ distribution between the membrane bilayers. Thus, we considered only the DPPC membrane. The results are presented in Table 3, featuring topological polar surface areas (polarity measure) calculated using MarvinSketch software, ChemAxon Ltd.

According to our in silico calculation, a rather small amount of coumarin 6 is able to saturate the membrane—1% (*w/w*) is the saturation level. Expectedly, the maximal amount of the dye transferred to liposomes, about 68%, was detected for PLGA-PEG-Cou6 NPs. Active dye leakage was also confirmed by confocal imaging for the double-stained PEG-PLGA-Cy5.5/Cou6 nanoparticles: the absence of co-localization for Cy5.5 and Cou6 in the 4T1 cells (see below, Section 3.7). Coumarin 6 is actively released from the nanoparticles and distributed in the cell membrane.

Rhodamine 123 also easily incorporates into the nanoparticles. However, it can just as easily leave it and then migrate through the membrane in both directions. Rho123 had little affinity to liposomes but was mostly transferred to the aqueous phase (Table 2). Rho 123 was the most unstable as a fluorescent label demonstrating a fast release and the subsequent staining of mitochondria (see Section 3.7.).

In the system of polylactide/dichloromethane, the affinity of any dye to PLGA is the same as its affinity to the solvent (log P_DCM/PLGA_ = 0). That means that when the solvent is being removed the dye molecules are distributed evenly in the nanoparticle matrix, and the maximum loading of the substance is limited by its solubility in dichloromethane (Table 3).

By their hydrophobicity, the dyes are arranged in the following order: DiI > Cou6 > Rho123 and, by their polarity, they are arranged in the opposite one: DiI < Cou6 < Rho123. The dyes’ affinity to polymers (polymer/water system, log P_PLGA/WAT_) and their affinity to lipid bilayers (log P_DPPC/WAT_) are arranged in the same order as hydrophobicity. The affinities of DiI to both polymers and lipids are much higher than those of coumarin 6, let alone rhodamine 123. In practice, this should mean that the probability of DiI leaking into water is rather low and is much lower compared to coumarin 6 and rhodamine 123.

### 3.6. Differential Scanning Calorimetry Assay of Interaction between Dyes and PLGA

The influence of DiI and coumarin 6 on PLGA thermal behavior was analyzed by differential scanning calorimetry (DSC). The dye-to-polymer ratio in the nanoparticle samples used for this assay was increased to 1:20 (*w/w*) to better reveal the influence of a dye on the polymer properties. As shown in Table 4, the glass transition temperatures (T_g_) of both labeled nanoparticles were lower compared with that of non-loaded PLGA NPs and a bulk polymer, which indicates that coumarin 6 and DiI act as plasticizers for PLGA.

### 3.7. In Vitro Behavior of PLGA-Cou6 Nanoparticles

Among the nanoparticles produced, several samples possessing the optimal physicochemical qualities as outlined previously were used in further in vitro experiments. A brief characterization of them is given in Table 5. The non-polymeric DiI formulation was used in this experiment to mimic the behavior of free dye in aqueous media when the nanoparticles are not properly separated from unbound dye. Surprisingly, the intracellular distribution patterns of free DiI (non-polymeric DiI formulation) and DiI encapsulated in the nanoparticles (PLGA-DiI) were similar (Figure 6).

**Figure 7:**
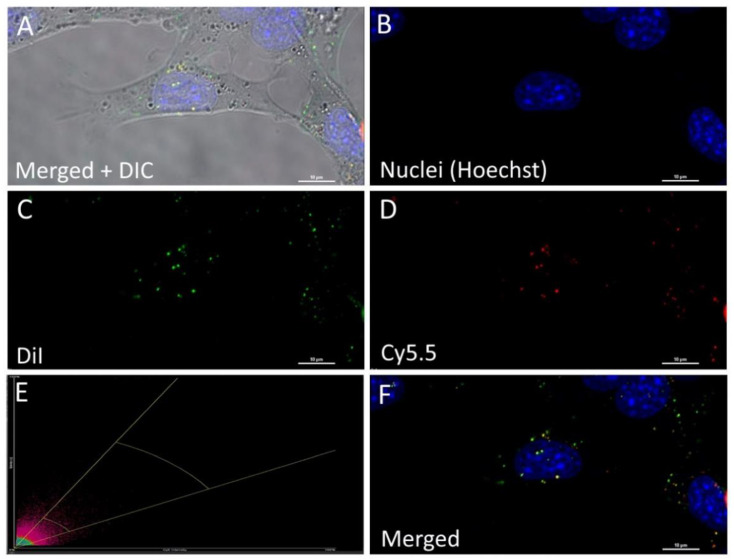
4T1 murine mammary carcinoma cells after 40 min incubation with the PLGA-Cy5.5/DiI nanoparticles: maximum intensity projection of a z-stack (Laser Scanning Confocal Microscopy, Nikon A1 MP). (**A**)—merged image and differential interference contrast (DIC); (**B**)—cell nuclei (Hoechst). (**C**)—DiI fluorescence; (**D**)—PLGA-Cy5.5 fluorescence; €—Scatterplot representing colocalization between DiI and Cy5.5 fluorescent signals. (**F**)—Merged image. Scale bar 10 μm.

**Figure 8:**
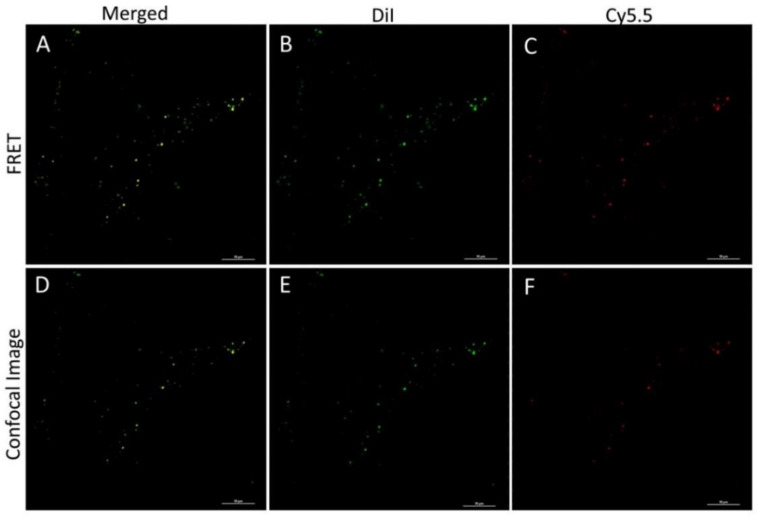
Imaging of 4T1 cells after 1 h of incubation with PLGA-Cy5.5/DiI NPs. Upper panels (**A**–**C**) represent the FRET signal at donor excitation wavelength (561 nm) -FRET phenomenon; lower panels represent DiI and Cy5.5 fluorescence separately (ex 561 and 647 nm); (**A**,**D**)—merged images; (**B**,**E**)—DiI fluorescence; (**C**,**F**)—Cy5.5 fluorescence. Laser scanning confocal microscopy (Nikon A1 MP). Scale bar 10 μm.

This phenomenon could be explained by the precipitation of the dye in the aqueous medium (PBS buffer), as was shown by molecular dynamic simulation. Interestingly, both, PLGA-DiI NP and non-polymeric DiI nanosuspension, appeared to be internalized into the cells without staining the cell membrane. Therefore, it was important to distinguish between the free dye nanoparticles and the PLGA nanoparticles to prevent the misinterpretation of the imaging data. Double-stained PLGA-Cy5.5/DiI nanoparticles were used for this purpose. However, as seen in Figure 7, both the DiI-labeled nanoparticles and the DiI nanosuspension displayed significant colocalization (Pearson’s correlation coefficient is 0.68; Mander’s overlap coefficient is 0.85) which suggests that, at this time, the DiI molecules are located mostly within the nanoparticles and, moreover, they are located close to the Cy5.5 molecules. The FRET signal in Figure 8 demonstrated that NP preserves their integrity during internalization process within 1 h of incubation. To determine the FRET signal, the samples were exited with a 561 nm laser (donor excitation wavelength), and the fluorescence was captured with both detectors simultaneously (both green and red fluorescent channels). Detectors from 570 to 620 nm and from 650 to 730 nm for DiI and Cy5.5 imaging, respectively, were used. Then the samples were exited with both 561 and 647 nm lasers, separately, for DiI and Cy5.5 excitation, respectively.

**Figure 9:**
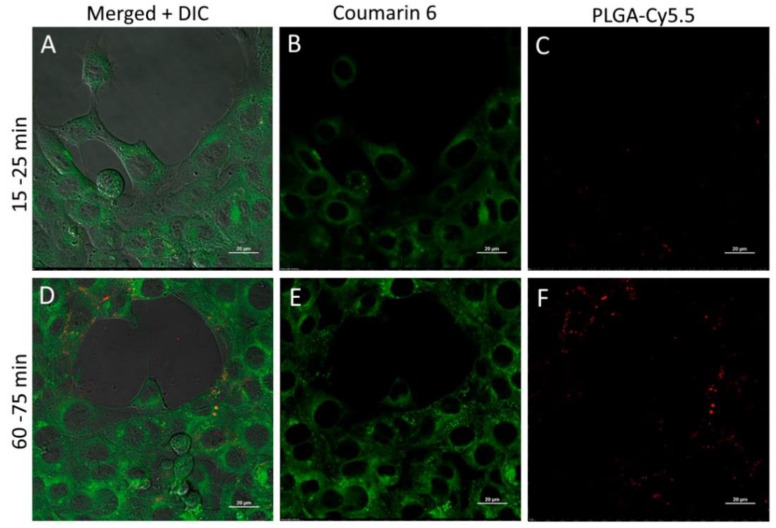
Internalization of PLGA-Cy5.5/Cou6 nanoparticles into 4T1 cells after 15–25 min (**A**–**C**) or 60–75 min (**D**–**F**) incubation (laser scanning confocal microscopy, Nikon A1 NP). (**A**,**D**)—merged image and differential interference contrast (DIC); (**B**,**E**)—coumarin 6 fluorescence; (**C**,**F**)—PLGA-Cy5.5 fluorescence. Scale bar 20 μm.

Coumarin 6 rapidly released from the double-stained nanoparticles and accumulated in the cells within the first minutes of incubation, while significant internalization of nanoparticles into the cells (traced by Cy5.5 signal) was observed only within 60 min of incubation (Figure 9).

**Figure 10:**
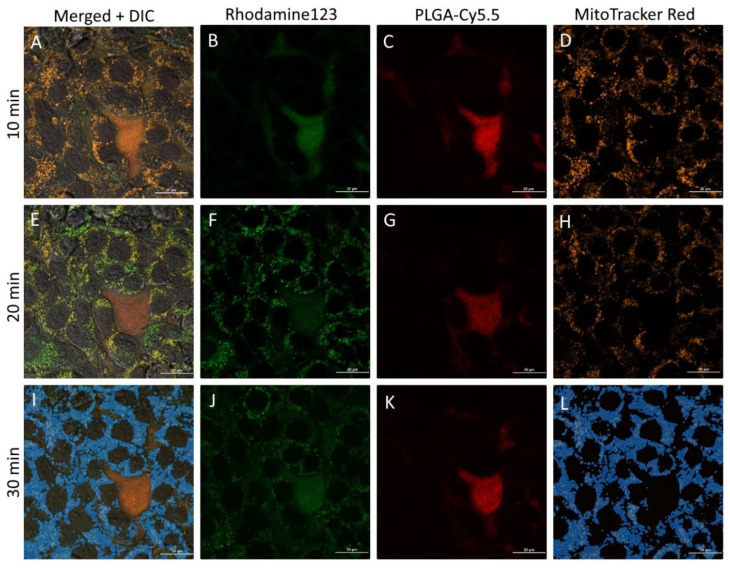

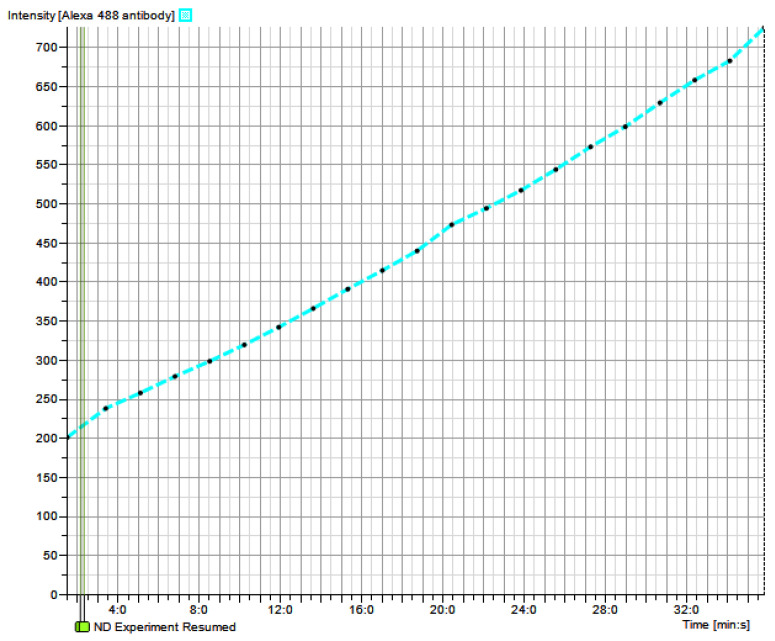
Imaging of 4T1 cells after 1 min (**A**–**D**) and 30 min (**E**–**H**) of incubation with PLGA-Cy5.5/Rh123 NPs. A,E—merged images and differential interference contrast (DIC); (**B**,**F**,**J**)—rhodamine 123 fluorescence; (**C**,**G**,**K**)—PLGA-Cy5.5 fluoresce; images (**I**,**L**) represent rhodamine 123 fluorescence intensity in the regions of mitochondrial fluorescence intensity (thresholding by mitochondrial fluorescence intensity of the images (**E**–**H**) was applied); (**M**)—Dynamic of rhodamine 123 accumulation within the mitochondria (ROIs are selected based on mitotracker fluorescence intensity—artificial blue channel (**I**,**L**)); 0–30 min of incubation. Laser scanning confocal microscopy (Nikon A1 NP). Scale bar 20 μm.

The same phenomenon was determined for rhodamine 123-loaded PLGA-Cy5.5 NP. However, rhodamine 123 penetrates into cells slower than coumarin 6, which correlates with computer simulation data (see Table 3). The dynamics of the nanoparticle and dye accumulation into the cells are shown in Figure 10. Moreover, rhodamine 123 was localized mainly in mitochondria (stained with MitoTracker Red, Life Technologies). The cells were imaged within 40 min of incubation with NPs. To track NP uptake by the cells and rhodamine 123 release and accumulation in real time, cells were not washed from the NP suspension. The graph in Figure 10M represents the increase in the fluorescence intensity of rhodamine 123 colocalized with mitochondria recognized in Figure 10K–O (applied blue channel). Rhodamine 123 accumulation was assessed in the cell mitochondria defined as regions of interest (ROI). ROI were obtained by thresholding based on the mitotracker fluorescence intensity profile.

### 3.8. In Vivo Behavior of PLGA-Cou6 Nanoparticles

**Figure 11:**
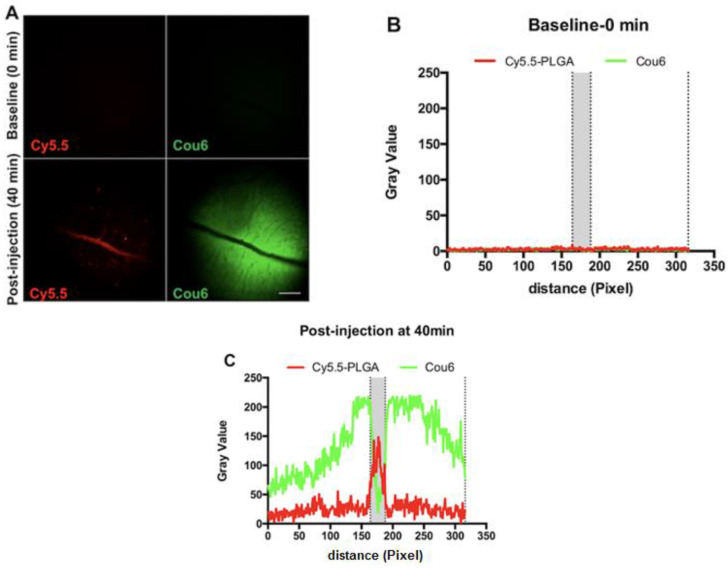
Biodistribution analysis of the intravenously injected Cy5.5-PLGA-Cou6 nanoparticles in rat retinal tissue. (**A**)—signal of Cou6 as encapsulated dye and Cy5.5 as covalently linked dye observed by the ICON technique before intravenous injection (baseline; upper row) or post injection at 40 min (lower row); scale bar: 400 μm. (**B**)—background intensity of signal of Cou6 and Cy5.5 before intravenous injection at baseline. (**C**)—intensity of signal of Cou6 and Cy5.5 40 min post injection. Area highlighted in gray in (**B**) and (**C**): blood vessel lumen.

To observe the in vivo distribution of Cou6 as the encapsulated label and Cy5.5 as a covalently linked label, the retinae of rats were imaged using in vivo confocal imaging after intravenous injection of the nanoparticles. As shown in Figure 11A, in the case of the Cy5.5-PLGA-Cou6 nanoparticles, the signal of Cy5.5 concentrated in the blood vessels (Figure 11A); however, the Cou6 signal was extremely bright in the tissue background of the retina, confirming the different distribution between the fluorescent labels encapsulated or covalently linked to the nanoparticles. The intensity analysis further confirmed that most of the Cy5.5 signals were visible inside the blood vessels, whereas the Cou6 signals were mainly distributed outside the blood vessel and in the tissue background.

In the case of Cou6 the in vivo neuroimaging (ICON) data for the PLGA-Cy5.5/Cou6 nanoparticles corresponded to the data obtained above: the nanoparticle signal (Cy5.5) was found in the retinal vessels, whereas the Cou6 signal was distributed in the tissue background. Retinal vessels are characterized by the presence of the blood–retinal barrier, which in many ways is similar to the blood–brain barrier [75]. The obtained results can be easily misinterpreted, creating an impression that the nanoparticles effectively cross the blood–retina barrier, when in fact, no signal from the core material is found beyond the vessels. This was expected, because it was shown [76] that coumarin 6 dye stains liposomes and cells with no significant release into aqueous media, and therefore, it would most likely leak in vivo. The in vivo behavior of DiI and Rh123 was described in our previous study [77]: Preliminary tests for PLGA nanoparticles loaded with DiI showed that the results depended on the amount of loosely-bound dye: washed DiI nanoparticles did not stain liposomes and cells, while non-washed did. DiI and rhodamine 123 colocalized with the cyanine5.5 signal well at early stages of the experiment; however, the kinetics of the signal fading was different, especially for rhodamine 123, which leaked from the nanoparticles and was eliminated quickly. In contrast to the in vitro experiments with liposomes, the intravenously injected DiI-labeled nanoparticles provided long-term staining of the endothelial cell membranes, even when the nanoparticles were washed. This discrepancy may be explained by a more effective contact of nanoparticles with blood vessel endothelial cells achieved in vivo that could be due to blood pressure, compared to the contact that was achieved using liposomes.

Dyes, especially hydrophobic dyes, are sometimes used as model drugs for the evaluation of the targeting capability of various delivery systems [78,79], which is due to the fact that they can be easily visualized unlike most drug molecules, which are rarely fluorescent. In this respect, the most important difference between drugs and labels is the requirement of their retention by the carrier. Indeed, the drug is supposed to be released sooner or later, whereas the label should be retained by the carrier as long as possible. Thus, in a recent study conducted by our group [80], coumarin 6 served as a model drug that, in concert with the above observations, was very loosely associated with the PLGA nanoparticles. However, being quickly released from the nanoparticles upon intravenous administration in mice, it was successfully delivered into the retina, whereas the nanoparticles remained associated with the blood capillary endothelial cells (Figure 11). Thus, while being not an optimal label, coumarin 6 helped to provide more evidence regarding the possibility of drug delivery by nanoparticles using a “kiss-and-run-mechanism”, wherein the drug is delivered into the tissue upon nanoparticle contact with the cell membrane, and no particle entry is necessary [81].

## 4. Conclusions

The findings of this study highlight the importance of the careful design and evaluation of the fluorescently labeled nanoparticles intended for biodistribution studies. Unsurprisingly, the most reliable way to label the nanoparticles is by the conjugation of the fluorescent dye to the polymeric core. In cases when studies require particles with physically entrapped dye, in vitro or in vivo experiments with the simultaneous tracking of the colocalization of both the nanoparticle and the dye have to be used to ascertain the nanoparticle system’s integrity and applicability.

We have shown that the usual threefold washing with water does not allow the removal of hydrophobic dyes from the surface of nanoparticles. In this case, the dye is practically not released into the usual model media but is being easily transferred from nanoparticles into the lipid bilayer of liposomes, which was also confirmed by the method of computer simulation.

Consequently, using the double-labeled nanoparticles as an example, it has been shown that, after the incubation of nanoparticles with coumarin 6, the latter is rapidly released from nanoparticles and stains the cell membranes of retinal vessels even after thorough preliminary washing of nanoparticles from free dye. Even very hydrophobic dyes included in the composition of polylactic nanoparticles and not covalently bound to their matrix can precipitate in a separate phase during the preparation of nanoparticles or be released from the surface of nanoparticles in biological media upon contact with lipid or protein components. The extent of this phenomenon depends on the affinity of the dye for the polymer matrix of the nanoparticles and the environmental conditions.

At this time we do not foresee that there is a single approach to traceable nanoparticles that can serve as a “golden standard” for all kinds of nanoparticle compositions and labels. Future studies should take into account the complex interferences of the fluorescent dye and the carrier prior to the use of fluorescently labeled nanoparticles.

## Figures and Tables

**Table 1:** Stability of labeling of the PLGA nanoparticles. In vitro release of DiI, Rh123, and Cou6 from PLGA nanoparticles (HSA, 1% solution in PBS, pH 7.4, 37 °C, *n* = 3).

Type of Nanoparticles	Fraction of Released Dye, %		
	1% Solution of Poloxamer 188	1% Solution of HSA	
	2 h	2 h	24 h
PLGA-DiI	9.8 ± 1.5	1.2 ± 0.1	42.3 ± 1.7
PLGA-Cy5.5/DiI	4.1 ± 0.3	<0.1	15.2 ± 0.7
PEG-PLGA-Cou6	17.0 ± 0.4	42.4 ± 2.1	65.0 ± 2.9
PLGA-Rh123	57.0 ± 3.5	n/d	n/d

**Table 2:** Dye partitioning in liposomes.

Type of Nanoparticles	Fraction of a Dye Transitioned to Liposomes, %	Amount of Dye Found in Liposomes, μg/mg
PLGA-Cou6	62.4 ± 7.1	0.93
PLGA-DiI	38.7 ± 0.6	1.45
PLGA-DiI *	0	0
PLGA-Rh123	8.1 ± 1.4	0.20
PLGA-Cy5.5	0	0

* Nanoparticles washed by centrifugation and subsequent GPC.

**Table 3:** Calculated parameters of the dyes’ distribution in different phases.

Parameter	DiI	Coumarin 6	Rhodamine 123
Topological polar surface area, Å^2^ (“polarity”)	6.25 *	42.43 *	91.48 *
log P_OCT/WAT_ ** (hydrophobicity)	10.3	5.0	0.04
log P_DPPC/WAT_ ** (affinity to a lipid bilayer)	8.6	5.2	−0.6
log P_DMPC/WAT_ ** (affinity to lipid bilayer)	8.7	5.2	−0.6
log P_PLGA/WAT_ ** (affinity to NPs’ polymeric matrix)	5.3	1.2	−3.6
log P_DCM/PLGA_ **	0	0	0
log P_DPPC/PLGA_ **	2.3	0.7	2.6
log P_DMPC/PLGA_ **	1.6	0.7	2.1

* Data calculated using MarvinSketch software, ChemAxon Ltd.; ** quantum mechanical calculation (25 °C, pH 7).

**Table 4:** Glass transition temperature (T_g_) of PLGA (bulk) and PLGA nanoparticles, both non-loaded and loaded with DiI or coumarin 6.

Sample	T_g_, °C
PLGA (bulk)	43.27
Non-loaded PLGA nanoparticles	44.90
Cou6-PLGA nanoparticles	43.91
DiI-PLGA nanoparticles	42.35

**Table 5:** Characteristics of fluorescent PLGA NPs used in in vitro experiments.

Type of NPs	Dye Content, µg/mg PLGA		EE, %	Mean Diameter Z-ave, nm	PDI	Zeta Potential, mV
PLGA-DiI	6.4		76.6	156 ± 5	0.156 ± 0.016	−27 ± 2
Non-polymeric DiI formulation	23.6 µg/mL *		-	333 ± 93	0.355 ± 0.077	−21 ± 6
PEG-PLGA/Cou6	27.2		99.7	139 ± 2	0.036 ± 0.028	−15 ± 3
Non-polymeric Cou6 formulation	32.6 µg/mL *		-	1086 ± 166	0.830 ± 0.111	−6.6 ± 0.1
PLGA-Cy5.5/DiI	DiI	6.2	77	137 ± 1	0.136 ± 0.022	−18 ± 1
	Cy5.5	0.8				
PEG-PLGA-Cy5.5/Cou6 NPs	Cou6	5.7	91	128 ± 1	0.137 ± 0.022	−22 ± 1
	Cy5.5	0.8				
PLGA-Cy5.5/Rh123	Rh123	6.8	96.6	98 ± 1	0.196 ± 0.031	−24 ± 4
	Cy5.5	0.8				

* For non-polymeric forms the dye content is given per ml of a suspension.

## Data Availability

Not applicable.

## Supplementary Materials

The following are available online at https://www.mdpi.com/article/10.3390/pharmaceutics13081145/s1, Table S1: Characteristics of fluorescent dyes; Table S2: Physicochemical parameters of the PLGA nanoparticles labeled with DiI, rhodamine 123 and Cy5.5 (representative samples); Table S3: Influence of the PEG-PLGA/PLGA ratio on Cou6-labeled nanoparticle size and the size distribution at the dye-to-polymer mass ratio of 1:170; Table S4: Influence of the Cou6-to-polymer ratio (*w/w*) on the size distribution of PEG-PLGA-Cou6 nanoparticles (PEG-PLGA/PLGA = 1:5, *w/w*, number of measurements = 4; mean ± SD); Table S5: Representative physicochemical parameters of the double-labeled PLGA nanoparticles; Table S6: Conformer distribution in liquid phases at 298.15 K and pH 7; Figure S1: Normalized spectra of the possible FRET pairs with Cy5.5 as acceptor. Top to bottom: rhodamine 123 (orange line) —Cy5.5 (blue line), DiI (purple line Cy5.5 (blue line), coumarin 6 (green line)—Cy5.5 (blue line). Absorbance spectra are in solid lines, and fluorescence spectra are in dashed lines. The spectral overlap between a donor absorbance spectrum and an acceptor excitation spectrum is an indication of the FRET efficiency. The efficiency of FRET is shown with filling. The overlap between DiI/Cy5.5 is the greatest of the three pairs; Figure S2: Fluorescence (solid line) and absorbance (dashed line) spectra of the PLGA-DiI/Cy5.5 nanoparticles. The arrow shows the direction of FRET; Figure S3: Fluorescence (solid line) and absorbance (dashed line) spectra of PLGA-Rh123/Cy5.5 NPs; Figure S4: Fluorescence (solid lines) and absorbance (dashed line) spectra of the PLGA-Cou6/Cy5.5 nanoparticles.
