# Supplementary material for: Fluorescently Labeled PLGA Nanoparticles for Visualization In Vitro and In Vivo: The Importance of Dye Properties
Source: Pharmaceutics. 2021 Jul 27;13(8):1145. doi: 10.3390/pharmaceutics13081145 (PMC8399891; doi:10.3390/pharmaceutics13081145)
Supplement: Supplementary file 1 [file pharmaceutics-13-01145-s001.zip › pharmaceutics-1286110-SM.pdf]

pharmaceutics

Supplementary Materials: Fluorescently Labeled PLGA Nanoparticles for Visualization In Vitro and In Vivo: The Importance of Dye Properties

Vasilisa Zhukova, Nadezhda Osipova, Aleksey Semyonkin, Julia Malinovskaya, Pavel Melnikov, Marat Valikhov, Yury Porozov, Yaroslav Solovev, Pavel Kuliaev, Enqi Zhang, Bernhard A. Sabel, Vladimir Chekhonin, Maxim Abakumov, Alexander Majouga, Jörg Kreuter, Petra Henrich-Noack, Svetlana Gelperina and Olga Maksimenko

Supplementary information

Synthesis of PLGA-Cy5.5

Polylactic-co-glycolic acid (PLGA, Resomer® RG 502 H, 3.0 g, ~631 μmol), 1-ethyl-3-(3-dimethylaminopropyl)carbodiimide hydrochloride (EDC, 12.7 mg, 66.2 μmol), N-hydroxysucccinimide (NHS, 7.6 mg, 66.0 μmol) were dissolved in methylene chloride, and Cyanine5.5 amine (5.0 mg, 6.6 μmol) and 30 μl (17.2 μmol) of N,N-diisopropylethylamine (DIPEA) were added to the solution (total methylene chloride volume was 50 ml). The reaction was allowed to proceed for 48 h at a room temperature with moderate stirring, protected from light. By-products of the reaction and an excess of the coupling reagents were removed by repeating washing with water and 1/1 water/methanol mixture (as Cyanine5.5 amine has a better solubility in alcohols than in water). The content of the dye in aqueous phase was monitored by spectrofluorimetry (samples were centrifuged prior to analysis in order to remove microdroplets of methylene chloride that might be present in aqueous phase). After washing, the organic phase was dried over anhydrous sodium sulfate and evaporated in vacuo. The residue was dissolved in 24 ml of ethyl acetate and the solution was added dropwise using a syringe to 240 ml of n-hexane which was vigorously stirred during the process. Flakes of polymer that formed were separated by filtration through a paper filter, collected, and dried under reduced pressure overnight. Formation of the PLGA-Cy5.5 conjugate was confirmed by a TLC method on silica gel-coated plates using a methylene chloride/methanol/water (6.5:2.5:0.4) mixture as eluent.

Stability of labeling after the nanoparticle preparation was verified by TLC performed as described above. The nanoparticles were dissolved in methylene chloride, and the undissolved colorless residue was filtered off using a syringe filter. The brightly colored filtrate was used for analysis.

Table S1. Characteristics of fluorescent dyes.

**Parameter**	**DiI**	**Coumarin 6**	**Rhodamine 123**	**Cyanine5.5 amine**
Chemical structure	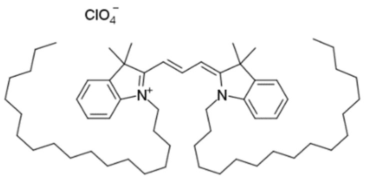	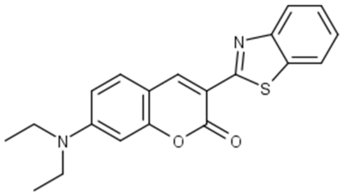	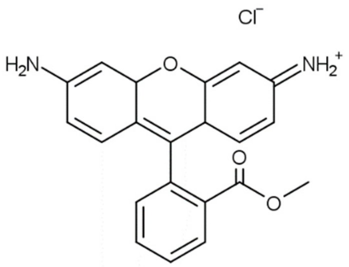	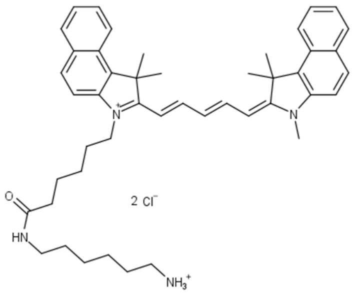
Molar mass, g/mol	933.89	350.44	380.83	753.88
Solubility in water#	Insoluble<br>(< 0.1 mg/ml)	Very slightly soluble<br>(< 1 mg/ml)	Slightly soluble<br>(< 10 mg/ml)	Slightly soluble<br>(< 10 mg/ml)
pKa*	2.56	4.20	0.73	-***
Fluorescence (max)**<br>λEX/λEM (nm)	551/566<br>549/565 (lipid membrane)	462/512 (ethanol)	507/527<br>511/534 (ethanol)<br>488/515-575 (PBS)	684/710 (ethanol)

According to the USP criteria (USP 38. General Notices and Requirements. Chapter 5.30. Description and solubility)
* Data calculated using Marvin Sketch ver. 20.14, ChemAxon Ltd.
** Manufacturers' data
*** pKa of Cyanine5.5 amine is not indicated because in all the samples it was covalently bound

Table S2. Physicochemical parameters of the PLGA nanoparticles labeled with DiI, rhodamine 123, and Cy5.5 (representative samples).

Nanoparticles	Initial dye-to-polymer ratio, *w/w*	Mean diameter, Z-Ave, nm	PDI	Zeta potential, mV	EE, %	Dye content, μg/mg
Non-labeled	-	113 ± 5	< 0.15	−30 ± 5	-	-
PLGA-DiI	1:155	86 ± 5	< 0.15	−39 ± 7	72	6.3
PLGA-Rh123	1:48.4	100 ± 1	< 0.20	−20 ± 2	96	5.0
PLGA-Cy5.5 (50% of PLGA-Cy5.5)	1:1200	119 ± 5	< 0.20	−22 ± 4	-	0.8

Optimisation of PLGA-Cou6 NPs using PEG

PEG molecules were introduced by using a mixture of PLGA and PEG-PLGA for the nanoparticle preparation. Addition of 20% w/w of PEG-PLGA led to the PDI decrease from 0.295 to 0.011 thus producing practically monodisperse nanoparticles (Table S3). In further experiments, the nanoparticles prepared from a (1:5) mixture of PEG-PLGA/PLGA were used (designated for simplicity as "PEG-PLGA" nanoparticles).

Polydispersity of the PEG-PLGA-Cou6 nanoparticles measured immediately after the preparation was found to be increasing in parallel with the amount of coumarin 6 used for nanoparticles preparation (Table S4). However, removal of the large particles or aggregates by centrifugation at 5000 rpm followed by filtration through the 0.45 μm filter led to a very considerable decrease of the polydispersity that was more considerable for the samples with a higher dye content.

The zeta potential also depended on the amount of coumarin 6 in the nanoparticles and decreased with the increasing of the dye-to-polymer ratios. Comparison of the non-labelled PEG-PLGA nanoparticles and a polymer-free dye nanosuspension (samples 5 and 6) shows that, in contrast to the non-labelled nanoparticles, the polymer-free dye suspension was prone to aggregation, exhibiting a PDI> 0.9 and a positive zeta-potential. There was no obvious correlation between the initial and final dye loading, probably due to the complexity of the system which led to non-proportional losses of the ingredients during the preparation and washing steps.

As was mentioned above, coumarin 6 is often used to track the nanoparticles fate because of its very strong fluorescent properties. Indeed, the encapsulation of Cou6 yielded the brightest nanoparticles, but this dye also strongly influenced the physicochemical properties of the PLGA, for example their aggregation stability. In the present study, aggregation of the PLGA-Cou6 nanoparticles could be reduced by hydrophilization of the surface with PEG;

Table S3. Influence of the PEG-PLGA/PLGA ratio on Cou6-labeled nanoparticle size and size distribution at the dye-to-polymer mass ratio of 1:170.

PEG-PLGA:PLGA ratio, w/w	Nanoparticle size and size distribution			
	Immediately after preparation		After freeze-drying and purification	
	Mean diameter Z-ave, nm	PDI	Mean diameter Z-ave, nm	PDI
1:0	161 ± 2	0.295 ± 0.020	122 ± 1	0.127 ± 0.011
1:5	143 ± 2	0.011 ± 0.005	131 ± 2	0.031 ± 0.023
1:10	211 ± 3	0.073 ± 0.035	150 ± 1	0.215 ± 0.012

Table S4. Influence of the Cou6-to-polymer ratio (*w/w*) on the size distribution of PEG-PLGA-Cou6 nanoparticles (PEG-PLGA/PLGA = 1:5, *w/w*, number of measurements = 4; mean±SD).

Sample	Initial Cou6-to-polymer ratio*, *w/w*	Cou6 content after preparation, μg/mg PLGA	Nanoparticle size and size distribution				Zeta potential, mV	
			Immediately after preparation		After centrifugation/filtration			
			Mean diameter, (Z-ave), nm	PDI	Mean diameter (Z-ave), nm	PDI	After preparation	After centrifugation/ filtration
1	1:170	15	143 ± 2	0.011 ± 0.005	144 ± 1	0.071 ± 0.012	−22.9 ± 0.4	−17.7 ± 2.3
2	1:81	26	134 ± 1	0.060 ± 0.016	148 ± 3	0.038 ± 0.025	−13.7 ± 0.6	−14.5 ± 2.6
3	1:43	25	208 ± 18	0.399 ± 0.028	134 ± 1	0.105 ± 0.026	−2.2 ± 1.9	−15.9 ± 0.6
4	1:25	33	155 ± 4	0.296 ± 0.030	139 ± 2	0.036 ± 0.028	−0.3 ± 0.2	−20.7 ± 0.4
5	0:100[#]	0	143 ± 2	0.168 ± 0.015	157 ± 1	0.171 ± 0.025	−31.9 ± 3.3	n/d
6	100:0[##]	-	1099 ± 76	0.971 ± 0.034	1086 ± 166	0.830 ± 0.111	+6.6 ± 0.1	n/d

* total amount of PLGA

# non-labeled PEG-PLGA nanoparticles

dye suspension (polymer-free)

Table S5. Representative physicochemical parameters of the double-labeled PLGA nanoparticles.

Nanoparticles	Average size and size distribution		Zeta potential, mV	EE, %	Dye content, μg/mg	
	Size, nm	PDI				
Non-labeled NPs	113±5	< 0.15	−30 ± 5	-	-	
PLGA-Cy5.5/DiI	129–137	0.081–0.136	−30 ± 5	73–77	Cy5.5<br>DiI	0.8<br>6.2–7.8
PEG-PLGA-Cy5.5/Cou6 (50% of PLGA-Cy5.5)	128	0.137	−22 ± 1	91	Cy5.5<br>Cou6	0.8<br>5.9
PLGA-Cy5.5/Rh123	98	0.196	−24 ± 4	97	Cy5.5<br>Rh123	0.8<br>6.8

FRET

FRET efficiency was used to estimate the interrelationship between DiI and Cy5.5 before imaging in vitro. Determination of FRET efficiency (E) was based on the difference between the quantum yield of fluorescence of donor in double-stained nanoparticles and the quantum yield of nanoparticles with the same encapsulated dye of the same amount was as follows:

$$E = 1 - \frac{QY_{da}}{QY_{d}}, \quad (1)$$

where QY_{da} is the quantum yield of DiI in the double-stained nanoparticles (PLGA-Cy5.5/DiI NPs), and QY_{d} is the quantum yield of donor (DiI) in the PLGA-DiI NPs. Measurements for FRET efficiency calculations were performed using λ_{EX} 526 nm.

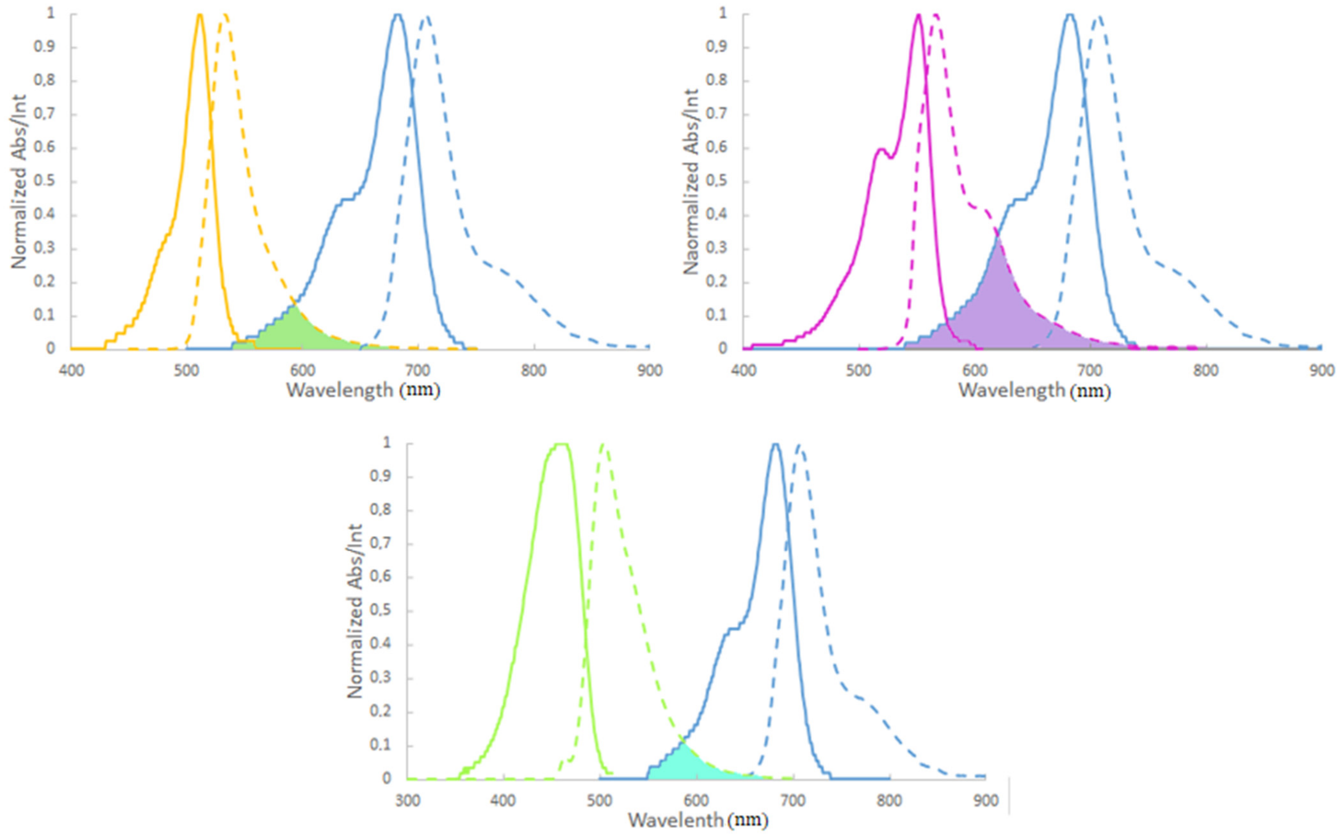

Figure S1. Normalized spectra of the possible FRET pairs with Cy5.5 as acceptor. Top to bottom: rhodamine 123 (orange line) —Cy5.5 (blue line), DiI (purple line Cy5.5 (blue line), coumarin 6 (green line) – Cy5.5 (blue line). Absorbance spectra are in solid lines, fluorescence spectra are in dashed lines. The spectral overlap between a donor absorbance spectrum and an acceptor excitation spectrum is an indication of the FRET efficiency the efficiency of FRET is shown with filling. Overlap between DiI/Cy5.5 is the greatest of the three pairs.

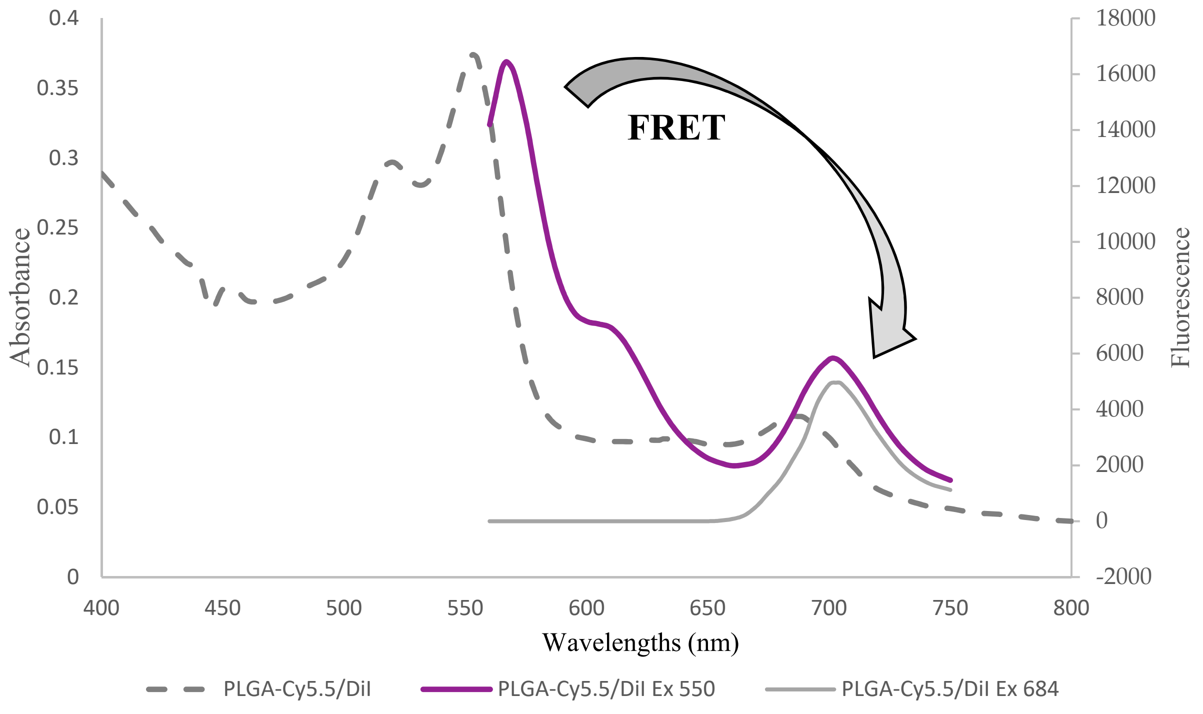

Figure S2. Fluorescence (solid line) and absorbance (dashed line) spectra of the PLGA-DiI/Cy5.5 nanoparticles. Arrow shows the direction of FRET.

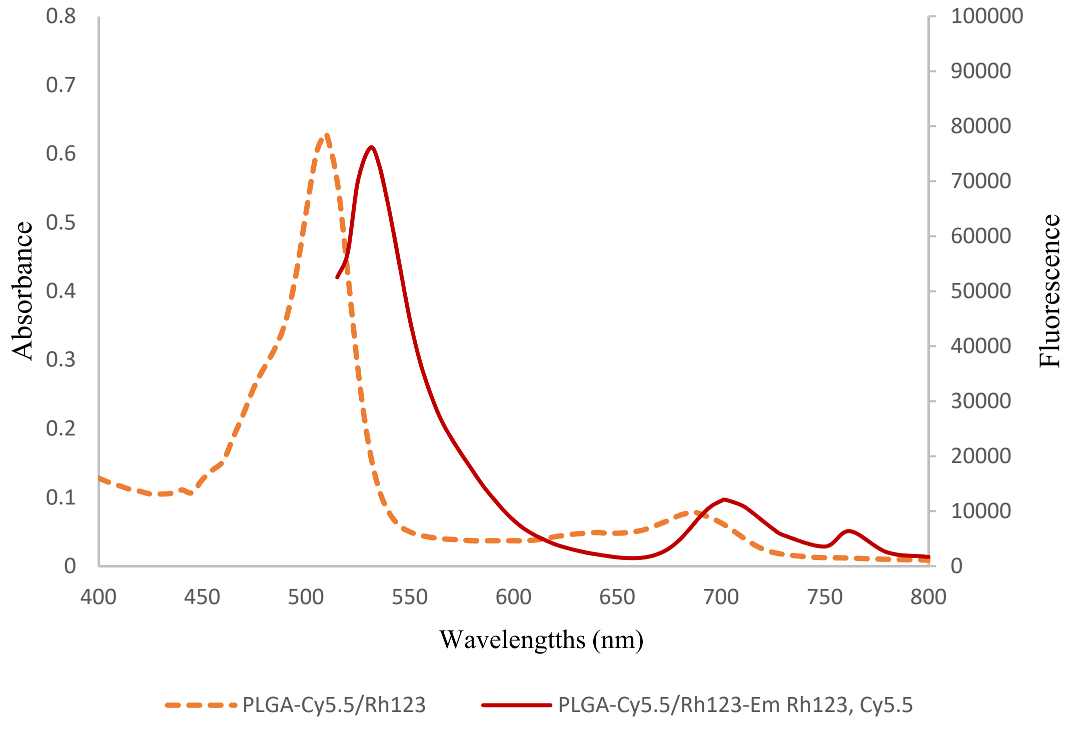

Figure S3. Fluorescence (solid line) and absorbance (dashed line) spectra of PLGA-Rh123/Cy5.5 NPs.

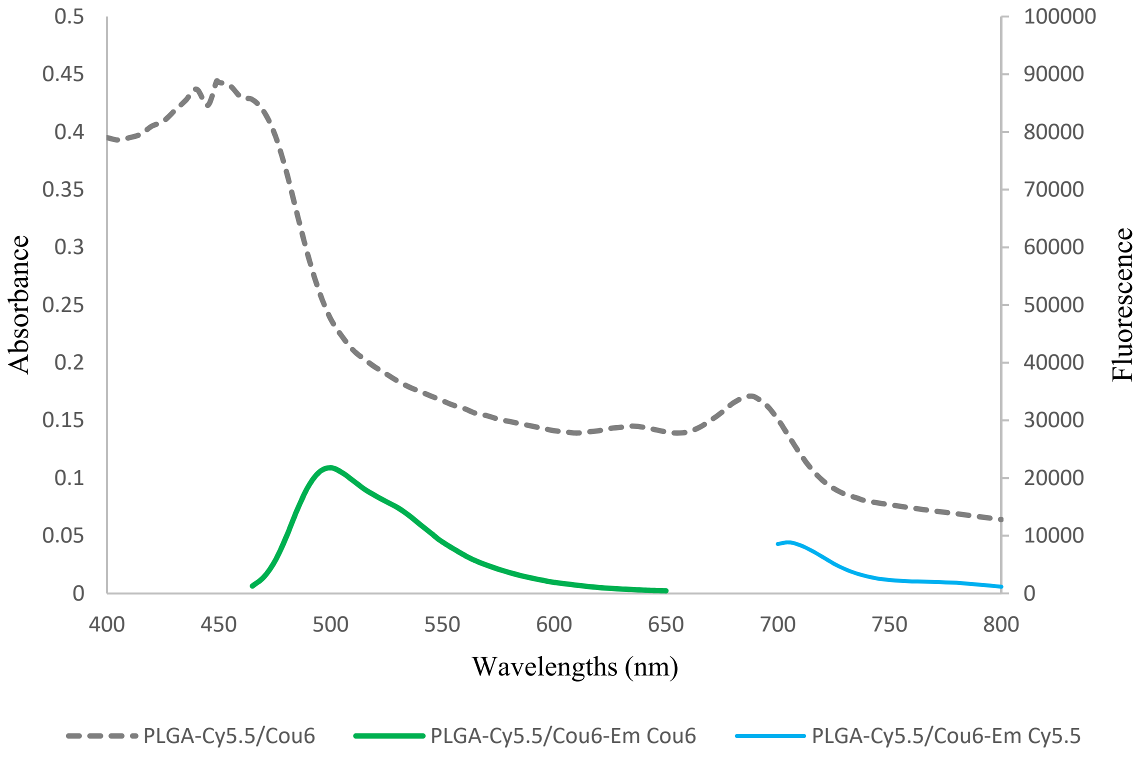

Figure S4. Fluorescence (solid lines) and absorbance (dashed line) spectra of the PLGA-Cou6/Cy5.5 nanoparticles.

Modelling of dye interaction with lipid membranes

Obtained results correlate well with the experimental data. Thus, it indicates that predicted conformers for each dye in various media reflect the real situation and the described calculation pipeline could be extended to variety of organic molecules to investigate their behavior in different liquids, polymers, and complex systems. No doubt that the chemical environment formed by various solvent systems will affect the relative conformer distribution (Table S6). The obtained results clearly demonstrated that in the real solutions the number of stable conformers is limited to a small number of structures. Although some of the considered molecules could be quite rigid, such as rhodamine 123 or coumarin 6, and others could be flexible like a DiI molecule, their stability allows them to exist in both polar and nonpolar solutions. However, specific interactions between solute and solvent molecules in solutions, it could be expected, that there will be no such conformer that is completely stable in one solvent and totally unstable in another one. Therefore, there is no reason to search for stable conformers in every considered system with different external parameters such as temperature and pressure, but it is rational to use only the limited list of conformers once obtained from the conformer search calculations instead. The relative stability of conformers could be used in the prediction of key thermodynamics and kinetic parameters, also in high-quality prediction of distribution coefficients, solubilities in liquids, and docking.

Table S6. Conformer distribution in liquid phases at 298.15 K and pH 7.

Compounds	Conformers	Prevalence in a given solvent, %		
		Water	Octanol-1	DPPC/DMPC
Rhodamine 123 cation	1	73.02	93.22	87.76
	2	19.00	4.57	8.17
	3	7.98	2.21	4.07
Coumarin 6	1	32.87	33.09	35.20
	2	32.68	32.34	32.71
	3	14.72	14.37	13.90
	4	19.73	20.20	18.19
DiI cation	1	33.37	63.76	59.64
	2	34.37	29.04	30.78
	3	4.39	5.82	7.32
	4	27.87	1.38	2.25
